# Hsp40 overexpression in pacemaker neurons delays circadian dysfunction in a *Drosophila* model of Huntington's disease

**DOI:** 10.1242/dmm.049447

**Published:** 2022-06-28

**Authors:** Pavitra Prakash, Arpit Kumar Pradhan, Vasu Sheeba

**Affiliations:** 1 Evolutionary and Integrative Biology Unit, Jawaharlal Nehru Centre for Advanced Scientific Research, Bangalore 560064, India; 2 Neuroscience Unit, Jawaharlal Nehru Centre for Advanced Scientific Research, Bangalore 560064, India

**Keywords:** Circadian, Heat shock protein, Hsp40, Huntingtin, Huntington's disease, Neurodegeneration, *Drosophila*, LNv

## Abstract

Circadian disturbances are early features of neurodegenerative diseases, including Huntington's disease (HD). Emerging evidence suggests that circadian decline feeds into neurodegenerative symptoms, exacerbating them. Therefore, we asked whether known neurotoxic modifiers can suppress circadian dysfunction. We performed a screen of neurotoxicity-modifier genes to suppress circadian behavioural arrhythmicity in a *Drosophila* circadian HD model. The molecular chaperones Hsp40 and HSP70 emerged as significant suppressors in the circadian context, with Hsp40 being the more potent mitigator. Upon Hsp40 overexpression in the *Drosophila* circadian ventrolateral neurons (LNv), the behavioural rescue was associated with neuronal rescue of loss of circadian proteins from small LNv soma. Specifically, there was a restoration of the molecular clock protein Period and its oscillations in young flies and a long-lasting rescue of the output neuropeptide Pigment dispersing factor. Significantly, there was a reduction in the expanded Huntingtin inclusion load, concomitant with the appearance of a spot-like Huntingtin form. Thus, we provide evidence implicating the neuroprotective chaperone Hsp40 in circadian rehabilitation. The involvement of molecular chaperones in circadian maintenance has broader therapeutic implications for neurodegenerative diseases.

This article has an associated First Person interview with the first author of the paper.

## Abstract (precis)

**Summary:** This study shows, for the first time, a neuroprotective role of chaperone Hsp40 in suppressing circadian dysfunction associated with Huntington's disease in a *Drosophila* model.

## INTRODUCTION

Huntington's disease (HD) is a neurodegenerative disease (ND) due to a dominant mutation in the huntingtin gene (*HTT*), leading to the expansion of the glutamine (Q) amino acid repeat tract in the huntingtin protein (HTT) beyond a threshold of 35-40 polyglutamine (polyQ) repeats. It shares several features with other NDs (Gusella and MacDonald, 2006; Bates et al., 2015), such as a typical middle-age onset, specificity of brain areas affected, motor and cognitive impairments, psychiatric disturbances, a progressive worsening with age, and decline in the quality of life and longevity. The presence of disease protein aggregates, aberrant proteostasis, synaptic toxicity, oxidative stress and neurodegeneration are shared pathophysiological mechanisms.

Circadian and sleep disruptions are now recognised as early symptoms of many NDs, including HD (Goodman et al., 2011; Morton et al., 2014; Lebreton et al., 2015; Bellosta Diago et al., 2017). The mammalian clock centre suprachiasmatic nucleus (SCN) is affected in HD mice, including molecular clock disruptions and reduction in the vasoactive intestinal peptide, a clock output neuropeptide (Morton et al., 2005; Maywood et al., 2010; Kudo et al., 2011; van Wamelen et al., 2013). Emerging evidence supports bi-directional crosstalk between the circadian and neurodegenerative axes, with circadian function impacting the aetiology and progression of NDs (Hood and Amir, 2017; Leng et al., 2019; Carter et al., 2021; Voysey et al., 2021). Improving clock function and sleep in HD mice have been neuroprotective (Pallier et al., 2007; Maywood et al., 2010; Ouk et al., 2017; Wang et al., 2017; Whittaker et al., 2018), whereas clock disruptions worsen ND (Krishnan et al., 2012; Lauretti et al., 2017; Kim et al., 2018; Sharma and Goyal, 2020).

Given the beneficial effects of circadian improvement on neurodegeneration, we aimed to uncover modifiers of expanded HTT (expHTT)-induced circadian arrhythmicity that could also serve as modifiers of neurodegenerative phenotypes. We have previously established and characterised a circadian model of HD in *Drosophila melanogaster* (Sheeba et al., 2008, 2010; Prakash et al., 2017), flies expressing an expHTT with 128 polyQ (HTT-Q128) in a subset of the pacemaker neurons, the ventral lateral neurons (LNv). The LNv express a critical circadian output neuropeptide, Pigment dispersing factor (Pdf), and are composed of the approximately four small LNv (sLNv) and four to five large LNv (lLNv) (Helfrich-Förster, 1995; Renn et al., 1999). Pdf and the sLNv are essential for locomotor activity/rest rhythms in constant darkness (DD) (Renn et al., 1999; Grima et al., 2004; Stoleru et al., 2004; Shafer and Taghert, 2009). Most flies expressing expHTT in the Pdf^+^ LNv (*Pdf>Q128*) exhibited disrupted behavioural activity rhythms in DD, a loss of Pdf from sLNv soma, loss of Period (Per) and its oscillations from LNv, and the presence of expHTT inclusions (Sheeba et al., 2008; Prakash et al., 2017). In these *Pdf>Q128* flies, we carried out a genetic screen for modifiers that could rescue circadian behavioural arrhythmicity. These genes are grouped under different categories based on function (Table S1), and the expressed proteins assist in neuronal function and are modifiers of neurodegeneration (Steffan et al., 2001; Gunawardena et al., 2003; Shulman and Feany, 2003; Sang et al., 2005; Wyttenbach and Arrigo, 2009; Zhang et al., 2009; Sutton et al., 2013; Kampinga and Bergink, 2016; Menzies et al., 2017; Metaxakis et al., 2018). Co-expression of expHTT in the LNv with candidates from the Heat shock protein (Hsp) or autophagy group significantly improved the rhythmicity of flies compared to that of their expHTT-only expressing counterparts (Table S1). In the Hsp group, these included *Hsp23*, *Hsp40* and *HSP70* homologues. The *tim>Q128,HSP70* also had better rhythmicity than *tim>Q128*. The central HSP70 and co-chaperone Hsp40 were chosen for further analysis.

Hsps play a central role in cellular proteostasis, aiding in protein folding, trafficking and degradation, and preventing aberrant interactions and disaggregation (Hartl and Hayer-Hartl, 2009; Kim et al., 2013; Nillegoda et al., 2018). Hsp40 and Hsp70 colocalise with mutant HTT aggregates (Jana, 2000; Muchowski et al., 2000; Kim et al., 2002; Scior et al., 2018), and their levels reduce with age, coinciding with proteostasis decline and middle-age HD onset (Hay, 2004; Ben-Zvi et al., 2009; Taylor and Dillin, 2011; Brehme et al., 2014; Hipp et al., 2019). Hsp upregulation alleviates proteotoxic stress and HD symptoms (Jana, 2000; Hansson et al., 2003; Branco et al., 2008; Labbadia et al., 2012; Brehme and Voisine, 2016; Kakkar et al., 2016), whereas their reduction aggravates neurodegenerative phenotypes in cell culture and animal models (Tagawa et al., 2007; Wacker et al., 2009; Hageman et al., 2010; Jiang et al., 2012; Scior et al., 2018). Therefore, the emergence of Hsps as modifiers in our screen is not surprising. However, the role of Hsps in circadian rehabilitation is relatively unexplored.

We investigated the role of Hsp40 and HSP70 as modifiers of expHTT-induced circadian neurodegenerative phenotypes in *Drosophila*. Of the two Hsps, Hsp40 emerged as the more potent modifier in delaying expHTT-induced phenotypes. Its overexpression postponed the loss of rhythmic locomotion over a substantial duration and the loss of Per and its oscillations from sLNv. Notably, there was a rescue of Pdf loss from sLNv and a decrease in the visible expHTT inclusion load favouring a new feature – a spot-like form of expHTT. HSP70 overexpression rescued rhythmicity and lowered expHTT inclusion numbers only at an early age, without rescuing Pdf or Per in the sLNv or affecting inclusions as the predominant form of expHTT in the LNv. Co-expression of Hsp40 and HSP70 in *Pdf>Q128* led to a synergistic improvement in the consolidation of activity rhythms. Overall, the present study establishes a role for Hsps as suppressors of expHTT-induced circadian impairments in a *Drosophila* circadian HD model.

## RESULTS

### Overexpression of Hsp40 or HSP70 delays arrhythmicity in flies expressing expHTT in LNv

**Fig. 1.**
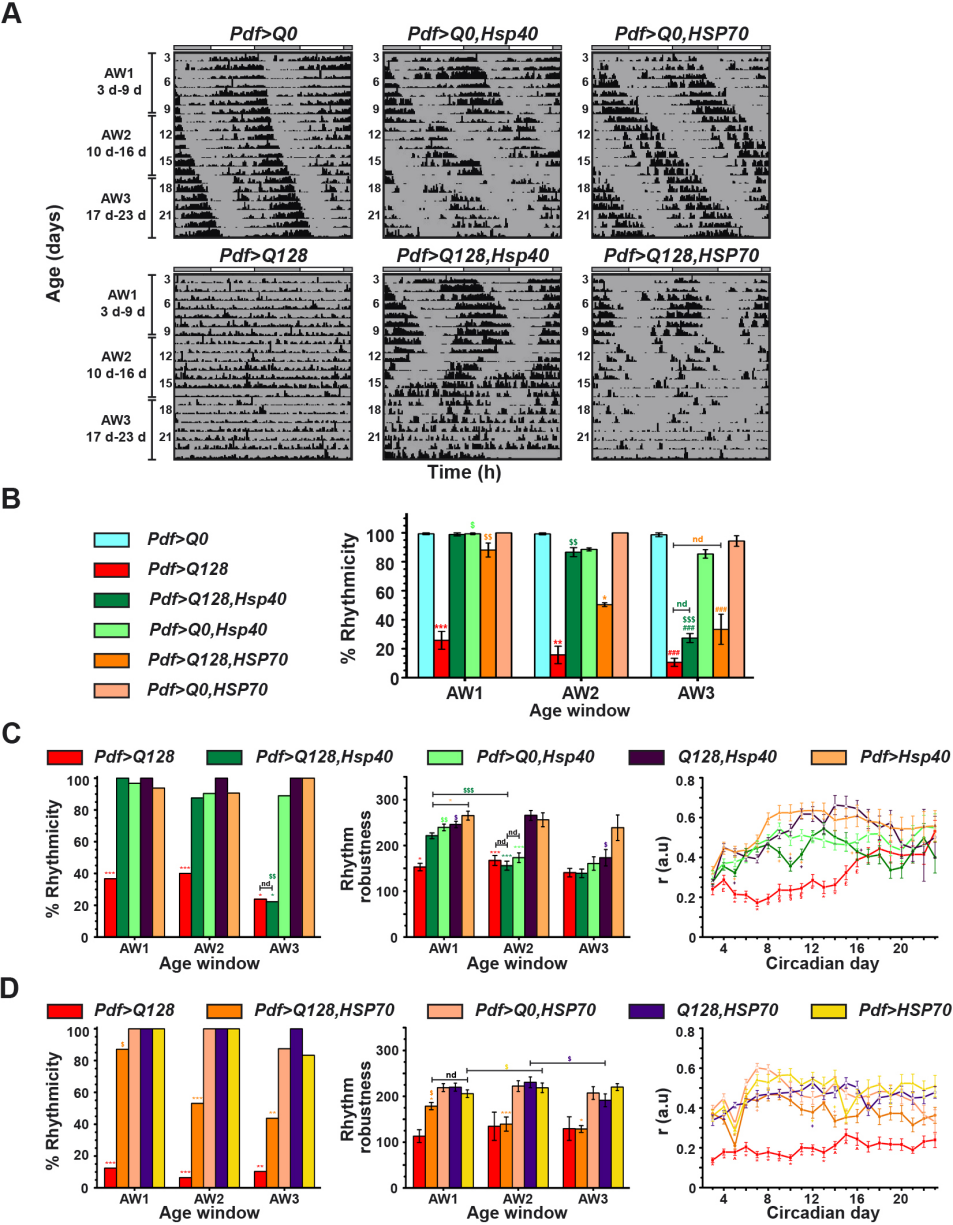
**In *Pdf>Q128* flies, Hsp40 overexpression leads to sustained behavioural rhythms, while HSP70 overexpression leads to early-age rhythmicity.** (A) Representative double-plotted actograms for flies showing activity data for 21 days (3-23 days) in constant darkness (DD) at 25°C for *Pdf>Q128*, *Pdf>Q128,Hsp40*, *Pdf>Q128,HSP70* and their respective *Q0* controls. Data of 21 days are divided into three 7 days age windows (AWs), shown on the left. The white and grey bars above actograms indicate the light and dark phases of prior 12 h:12 h light: dark cycles (LD). (B) The percentage rhythmicity averaged over at least three independent runs is plotted across AWs. (C,D) Comparison of percentage rhythmicity (left), mean rhythm robustness (middle) and mean ‘*r*’ value (right) between genotypes across age, with the main experimental genotype being *Pdf>Q128,Hsp40* (C) and *Pdf>Q128,HSP70* (D). a.u., arbitrary units. *Pdf>Q128* was not considered for between-genotype statistical comparisons of robustness across AWs in the HSP70 overexpression experiment (D, middle) and during AW3 in the Hsp40 overexpression experiment (C, middle), as very few flies were rhythmic. Also, for AW3, *Pdf>Q128,Hsp40* in the Hsp40 overexpression experiment was not considered for statistical analysis of robustness. Across all panels, coloured symbols represent statistically significant differences: coloured ‘*’ indicates age-matched differences of the respective coloured genotype from all other genotypes or indicated genotype; coloured ‘#’ indicates age-matched differences from all *Q0*-containing controls; and coloured ‘$’ indicates differences across age for the respective-coloured genotype. Statistical significance represented by symbols: single, *P*<0.05; double, *P*<0.01; triple, *P*<0.001. nd, not different. For the panels of ‘*r*’ values (C, right, D, right), red-coloured symbols indicate significant differences at *P*<0.05 for *Pdf>Q128* from ‘*’ all other genotypes, ‘§’ from all genotypes except *Pdf>Q128,Hsp40*, ‘∧’ from all genotypes except *Pdf>Q128,HspP70*, ‘£’ from all non-expanded controls, and orange ‘*’ for *Pdf>Q128,HSP70* from all other genotypes. Coloured ‘+’ near the error bar of a data point indicates significant differences at *P*<0.05 of the respective-coloured genotype from the data-point genotype. Error bars are s.e.m. *n* for these analyses are shown in Table S2: under experiment 1 for genotypes of C, under experiment 1 for *HSP70*-related genotypes of D, and experiment 4 for *Pdf>Q0* and *Pdf>Q128* of D, and all independent experiments considered for genotypes of B.

Flies expressing expHTT in LNv are arrhythmic immediately upon entering DD at 25°C (Fig. 1A, bottom left). Overexpressing Hsp40 or HSP70 in *Pdf>Q128* flies delayed the onset of this arrhythmicity (Fig. 1A, bottom middle and right). Most flies co-expressing HTT-Q128 with Hsp40 were rhythmic during the early age window (henceforth, AW1) and mid-age window (henceforth, AW2), their mean rhythmicities being comparable to those of controls expressing HTT-Q0 and significantly higher than those of *Pdf>Q128* (Fig. 1B). However, rhythmicity of *Pdf>Q128,Hsp40* during a later age window (henceforth, AW3) declined significantly more than during the earlier AWs and when compared to their age-matched controls and was like that of *Pdf>Q128*. Despite a drop in rhythmicity compared to AW1, most *Pdf>Q128,Hsp40* flies remained rhythmic during AW2. *Pdf>Q128,HSP70* had significantly higher rhythmicity than *Pdf>Q128* in AW1 and AW2 (Fig. 1B). However, it was like controls only in AW1, beyond which it declined. Notably, in AW2, although ∼50% of *Pdf>Q128,HSP70* remained rhythmic, their rhythmicity was significantly lower than that of *Pdf>Q128,Hsp40*. In AW3, like *Pdf>Q128,Hsp40* and *Pdf>Q128, Pdf>Q128,HSP70* also had poor rhythmicity. Whereas the rhythmicity of *Pdf>Q128,Hsp40* was like that of background controls across AW1 and AW2 (Fig. 1C, left; Fig. S2A, middle column), that of *Pdf>Q128,HSP70* was control-like only during AW1 (Fig. 1D, left; Fig. S2A, right column). Unlike the sharp fall in rhythmicity of *Pdf>Q128,HSP70* in AW2, *Pdf>Q128,Hsp40*, despite having a progressive reduction in rhythmicity with age, showed a significant fall in mean rhythmicity only in AW3 (Fig. 1B,C, left, D, left). Thus, the rescue of rhythmicity by Hsp40 lasted longer than that by HSP70.

In AW1, the rhythmic flies of *Pdf>Q128,Hsp40* had robust rhythms comparable to those of most controls and with significantly higher robustness than *Pdf>Q128* (Fig. 1C, middle). In AW2, the robustness of rhythmicity of *Pdf>Q128,Hsp40* dropped lower than that of both parental controls and was comparable to that of *Pdf>Q128*. Overall, the overexpression of Hsp40 in *Pdf>Q128* flies rescued both rhythmicity and rhythm robustness in AW1. In AW1, the rhythmic *Pdf>Q128,Hsp40* and *Pdf>Q0,Hsp40* flies had longer periods than other controls (Fig. 1A; Fig. S2A,B). The *Pdf>Q128,Hsp40* flies had significantly better activity consolidation than *Pdf>Q128* flies across ages (5-8 days and 11-14 days) (Fig. 1C, right). However, this improved consolidation was comparable to that of controls at 6-7 days and 12-15 days. Thus, overexpression of Hsp40 in *Pdf>Q128* flies rescues both rhythmicity and rhythm strength at an early age. Activity rhythms persist until the middle age, postponing arrhythmicity onset by more than 2 weeks. These results indicate that Hsp40 is a potent suppressor of expHTT-induced circadian behavioural arrhythmicity.

The rhythmic flies of *Pdf>Q128,HSP70* exhibited weaker rhythms than those of controls across AWs, and robustness in older AWs was lower than that in AW1 (Fig. 1D, middle). Controls *Pdf>Q0,HSP70* and *Pdf>HSP70* mostly had longer periods than rhythmic *Pdf>Q128,HSP70* and *Q128,HSP70* across AWs (Fig. 1A; Fig. S2A,C). The activity consolidation of *Pdf>Q128,HSP70* was significantly better than that of *Pdf>Q128* across 4-5 days and 6-13 days, and was comparable to that of controls at most of these ages (Fig. 1D, right). Thus, the overexpression of HSP70 in *Pdf>Q128* flies improves their early-age rhythmicity and activity consolidation. In contrast to the rescue with Hsp40 overexpression, rhythm rescue with HSP70 overexpression at an early age did not extend to the middle age. Hence, HSP70 is a partial suppressor of expHTT-induced circadian behavioural arrhythmicity and is less efficient than Hsp40.

### Co-expression of Hsp40 and HSP70 synergistically improves the consolidation of activity rhythms in flies expressing expHTT in LNv

**Fig. 2.**
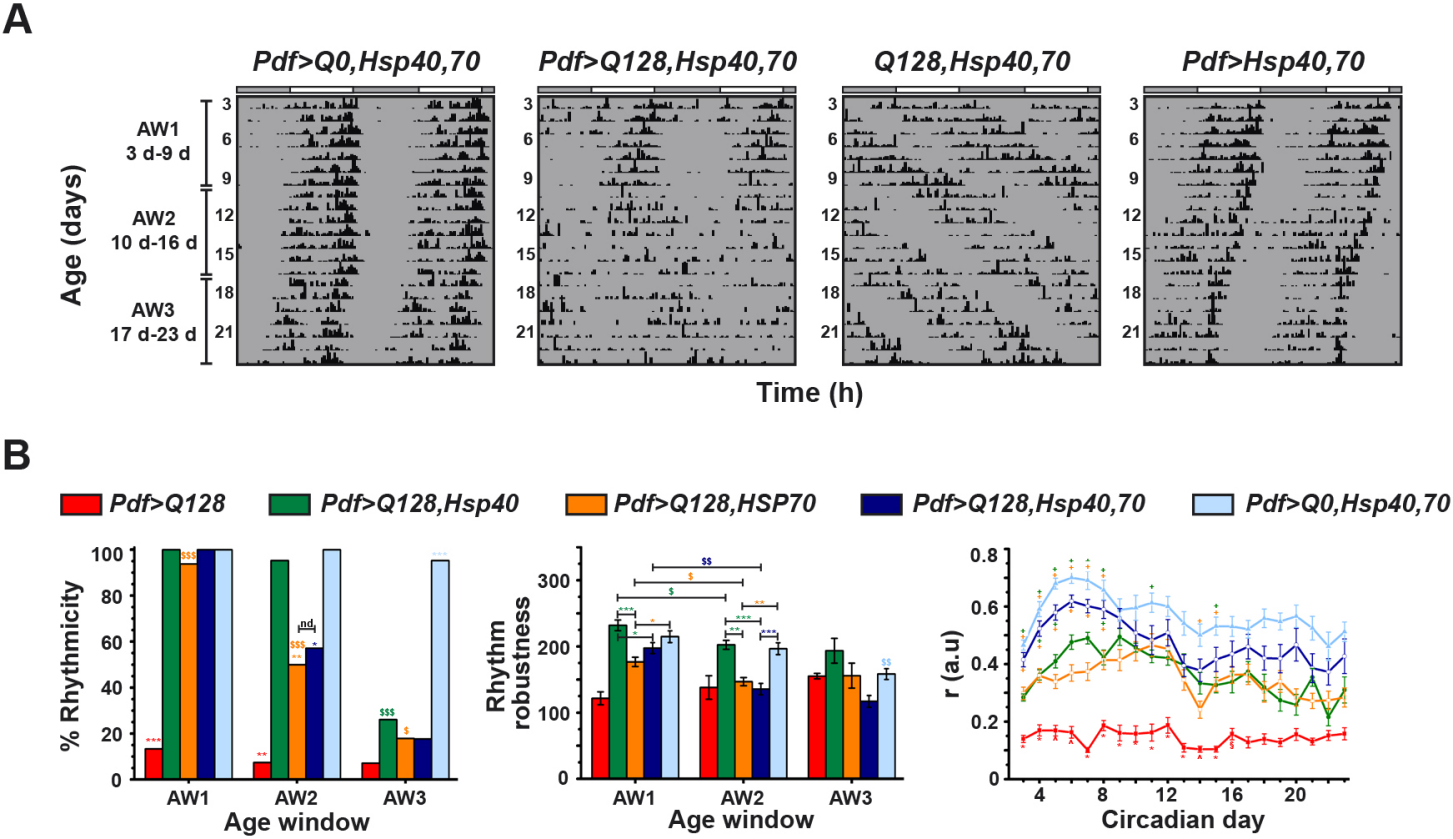
***Pdf>Q128* flies co-expressing both Hsp40 and HSP70 have greater daily activity consolidation than those expressing either Hsp expressed alone.** (A) Representative double-plotted actograms for flies showing activity data for 21 days (3-23 days) in DD at 25°C for *Pdf>Q128,Hsp40,70* and its controls. (B) The percentage of rhythmic flies (left), mean rhythm robustness (middle) and mean ‘*r*’ value (right) comparing genotypes across age. *Pdf>Q128* and AW3 are omitted from between-genotype statistical tests for robustness. Data post-16 days are omitted from between-genotype statistical tests of ‘*r*’ due to very few surviving flies. a.u., arbitrary units. Across all panels, coloured symbols represent statistically significant differences: coloured ‘*’ indicates age-matched differences of the respective-coloured genotype from all other genotypes or indicated genotype; coloured ‘#’ indicates age-matched differences of the respective-coloured genotype from all *Q0*-containing controls; and coloured ‘$’ indicates differences across age for the respective-coloured genotype. Statistical significance represented by symbols: single, *P*<0.05; double, *P*<0.01; triple, *P*<0.001. nd, not different. For the panel of ‘*r*’ values (B, right), red-coloured symbols indicate significant differences at *P*<0.05 for *Pdf>Q128* from ‘*’ all other genotypes, ‘§’ from all genotypes except *Pdf>Q128,Hsp40*, ‘∧’ from all genotypes except *Pdf>Q128,HSP70*, and orange ‘*’ for *Pdf>Q128,HSP70* from all other genotypes. Coloured ‘+’ near the error bar of a data point indicates significant differences at *P*<0.05 of the respective-coloured genotype from the data-point genotype. Error bars are s.e.m. *n* for these analyses are shown under the synergistic effect experiment of Table S2.

Previous studies show that co-expression of Hsp40 and Hsp70 has a synergistic effect of providing a greater effect on neurodegenerative features than expressing Hsp40 or Hsp70 individually (Chan et al., 2000; Jana, 2000; Kobayashi et al., 2000; Muchowski et al., 2000; Sittler et al., 2001; Bailey et al., 2002; Bonini, 2002; Rujano et al., 2007). Therefore, we asked whether overexpression of both Hsps provides a greater rescue (e.g. sustained robust rhythms across AWs) than expressing each alone. In AW1, flies expressing both Hsps in the presence of expHTT, i.e. *Pdf>Q128,Hsp40,70* were mostly rhythmic, comparable to *Pdf>Q128,Hsp40*, *Pdf>Q128,HSP70* and control *Pdf>Q0,Hsp40,70* and significantly better than *Pdf>Q128* (Fig. 2A,B, left). However, in AW2, the percentage rhythmicity of *Pdf>Q128,Hsp40,70*, like that of *Pdf>Q128,HSP70*, dropped significantly compared to AW1, while remaining higher than that of *Pdf>Q128*, but lower than that of *Pdf>Q128,Hsp40* and *Pdf>Q0,Hsp40,70* (Fig. 2B, left). In AW3, the rhythmicity percentage of *Pdf>Q128,Hsp40,70* declined further and, like for single Hsp overexpression, was comparable to that of *Pdf>Q128*. In AW1, the rhythmic *Pdf>Q128,Hsp40,70* flies exhibited robust rhythms comparable to those of control *Pdf>Q0,Hsp40,70* and the single rescue *Pdf>Q128,HSP70* (Fig. 2B, middle). Its period was similar to that of most other genotypes across age (Fig. 2A; Fig. S2D). Both *Pdf>Q128,Hsp40,70* and *Pdf>Q128,HSP70* had weaker rhythms than *Pdf>Q128,HSP70* in AW1 and AW2 and than *Pdf>Q0,Hsp40,70* in AW2 (Fig. 2B, middle). Interestingly, the extent of activity consolidation ‘*r*’ of *Pdf>Q128,Hsp40,70* was significantly greater than that of *Pdf>Q128* and both *Pdf>Q128,Hsp40* and *Pdf>Q128,HSP70* during most of the early age, while remaining comparable to that of *Pdf>Q0,Hsp40,70* and *Pdf>Hsp40,70* (Fig. 2B, right; Fig. S2E). Overexpression of both Hsp40 and HSP70 (*Pdf>Hsp40,70*) leads to a higher ‘*r*’ and, by extension, better-consolidated activity rhythms than those of experimental genotypes of *Pdf>Q128*, *Pdf>Q128,Hsp40* and *Pdf>Q128,HSP70* in early and middle ages, and also from control *Q128,Hsp40,70* at early ages (Fig. S2E). This enhanced consolidation upon expressing both the Hsps in LNv is also reflected in the significantly higher ‘*r*’ of *Pdf>Q0,Hsp40,70* and *Pdf>Q128,Hsp40,70* than that of the other experimental genotypes. Thus, although overexpression of both Hsp40 and HSP70 in *Pdf>Q128* did not have a synergistic effect on percentage rhythmicity per se, there was a synergistic improvement in early-age activity consolidation.

### Hsp40 overexpression in flies expressing expHTT in LNv rescues Pdf^+^ sLNv soma numbers

**Fig. 3.**
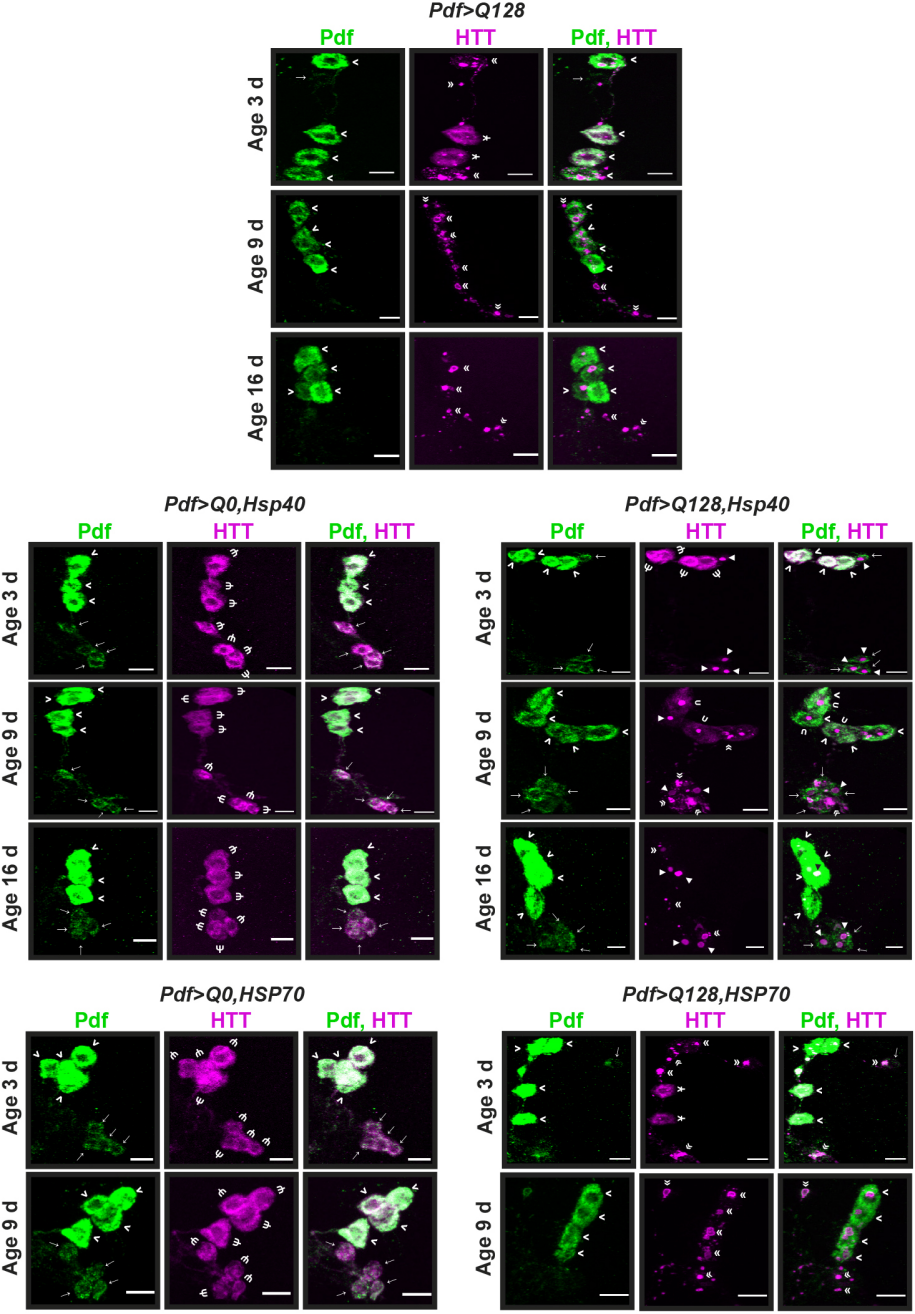
***Pdf>Q128* flies overexpressing Hsp40 retain Pdf^+^ small ventrolateral neuron (sLNv) soma across age.** Representative images of adult fly brains stained for Pdf (green) and HTT (magenta) in ventrolateral neurons (LNv) at 3 days, 9 days and 16 days for *Pdf>Q128* (top panel sets), *Pdf>Q0,Hsp40* (middle-left panel sets), *Pdf>Q128,Hsp40* (middle-right panel sets); and at 3 days and 9 days for *Pdf>Q0,HSP70* (bottom-left panel sets) and *Pdf>Q128,HSP70* (bottom-right panel sets). Indicated in the images are sLNv soma (‘→’), large ventrolateral neuron (lLNv) soma (‘>’), Diff expanded HTT (expHTT) (‘Ψ’), Diff+Inc expHTT (‘Ұ’), Diff+Spot expHTT (‘υ’), Spot expHTT (‘◄’) and Inc expHTT (‘«’) for the five genotypes. Scale bars: 10 µm.

**Fig. 4.**
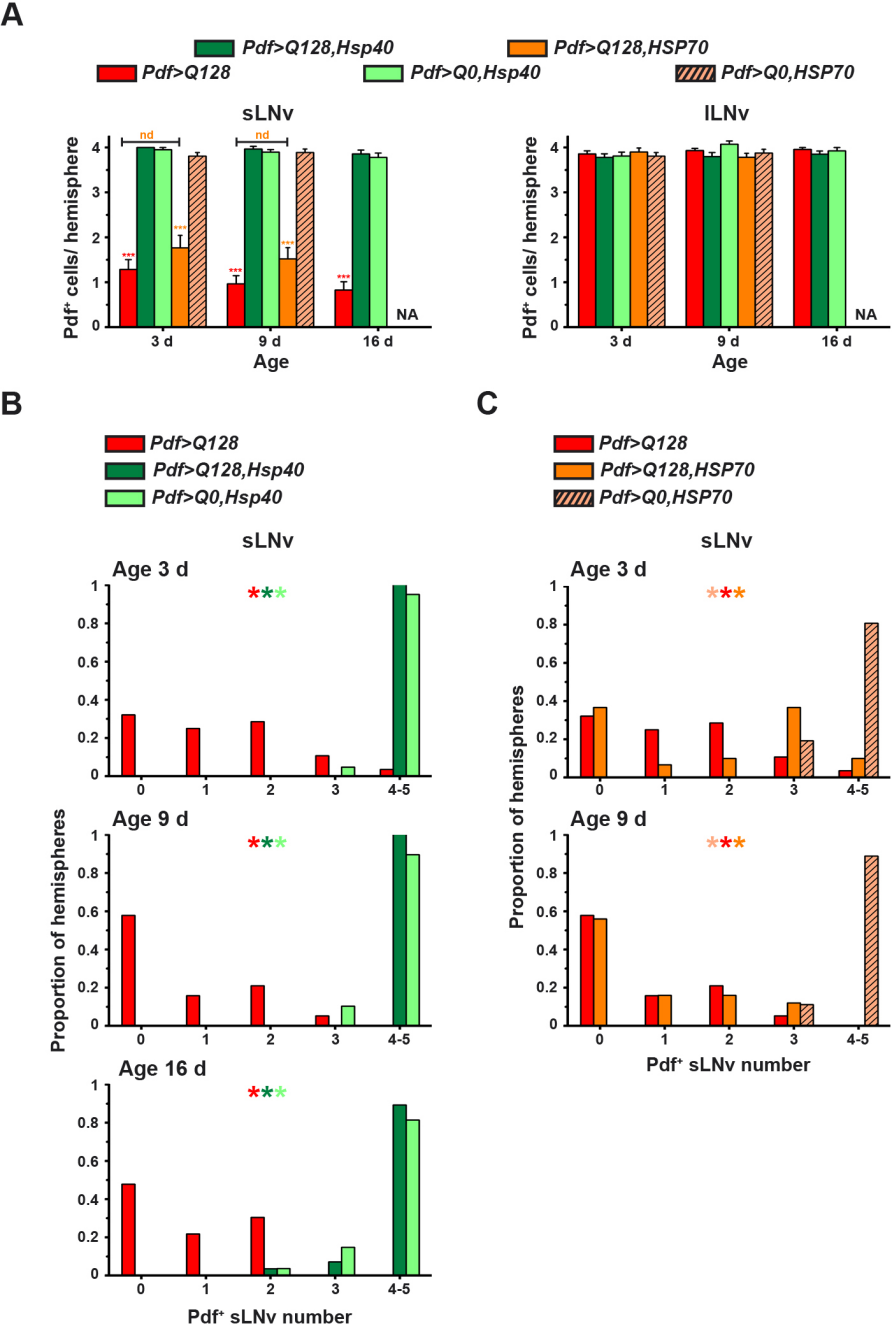
***Pdf>Q128* flies overexpressing Hsp40 have control-like Pdf^+^ sLNv soma numbers.** (A) Mean number of Pdf^+^ sLNv soma (left) and lLNv soma (right) across three ages. Symbols indicate significant differences, ‘*’ for age-matched, inter-genotype differences and ‘$’ for differences between ages for each genotype: red ‘*’ for *Pdf>Q128* from all other genotypes except *Pdf>Q128,HSP70*, and orange ‘*’ for *Pdf>Q128,HSP70* from other genotypes except *Pdf>Q128*, at *P<0.05, **P<0.01, ***P<0.001. nd, not different. NA, not applicable; because early-age-rescue of Pdf^+^ LNv was not seen in *Pdf>Q128,HSP70*, the dissections at 16 days were not done for them. (B,C) Frequency distribution of the proportion of hemispheres with 0-5 Pdf^+^ sLNv soma numbers, comparing *Pdf>Q128* with *Pdf>Q128,Hsp40* and *Pdf>Q0,Hsp40* at 3 days, 9 days and 16 days (B), and with *Pdf>Q128,HSP70* and *Pdf>Q0,HSP70* at 3 days and 9 days (C). Multiple coloured ‘*’ indicates significantly different distribution shapes between genotypes, with the first colour of the reference genotype and the subsequent colours of genotypes differing from the reference at *P*<0.01. Error bars are s.e.m. *n* for these analyses are shown in the top-left cell sets of Table S3.

We then investigated whether overexpression of Hsp40 or HSP70 in *Pdf>Q128* also rescues LNv cellular features. As described previously (Sheeba et al., 2010; Prakash et al., 2017) and as is also shown here, *Pdf>Q128* flies had a loss of Pdf from the sLNv soma from an early age, whereas Pdf in lLNv soma was unaltered (Fig. 3, top panel set; Fig. 4A). In contrast, flies overexpressing Hsp40, the *Pdf>Q128,Hsp40*, showed significantly higher Pdf^+^ sLNv soma numbers than *Pdf>Q128* and were indistinguishable from control *Pdf>Q0,Hsp40* across ages (Fig. 3, middle panel sets; Fig. 4A, left). The shapes of the frequency distributions of Pdf^+^ sLNv numbers for *Pdf>Q128,Hsp40* across age were left-skewed, like those of controls, with most hemispheres having four to five sLNv, and differed significantly from the right-skewed distribution of *Pdf>Q128* (Fig. 4B). In contrast, at 3 days and 9 days, the Pdf^+^ sLNv soma numbers of *Pdf>Q128,HSP70* were diminished, like those of *Pdf>Q128* and significantly lower than those of control *Pdf>Q0,HSP70* and *Pdf>Q128,Hsp40* (Fig. 3, bottom panel sets; Fig. 4A, left). Mirroring the mean Pdf^+^ sLNv soma numbers was the shape of their distributions at both ages: *Pdf>Q128,HSP70* was like *Pdf>Q128* and different from controls (Fig. 4C). The Pdf^+^ lLNv soma numbers were comparable for all the genotypes across age (Fig. 3, ‘>’; Fig. 4A, right). Thus, overexpression of Hsp40, but not HSP70, completely rescues Pdf^+^ sLNv numbers. The sustained circadian rhythm rescue in *Pdf>Q128,Hsp40* accompanied by the rescue of the circadian output neuropeptide Pdf in the soma of pacemaker neurons sLNv suggest Hsp40 as a disease modifier effective in restoring cellular function as well as associated behaviour. The lack of rescue of Pdf^+^ sLNv soma in *Pdf>Q128,HSP70* at an early age despite the persistence of circadian activity rhythms suggests an unconventional mode of rhythm restoration by HSP70 in the absence of somal Pdf in the sLNv.

### Hsp40 overexpression in *Pdf>Q128* flies reduces the inclusion form of expHTT in favour of a new form

**Fig. 5.**
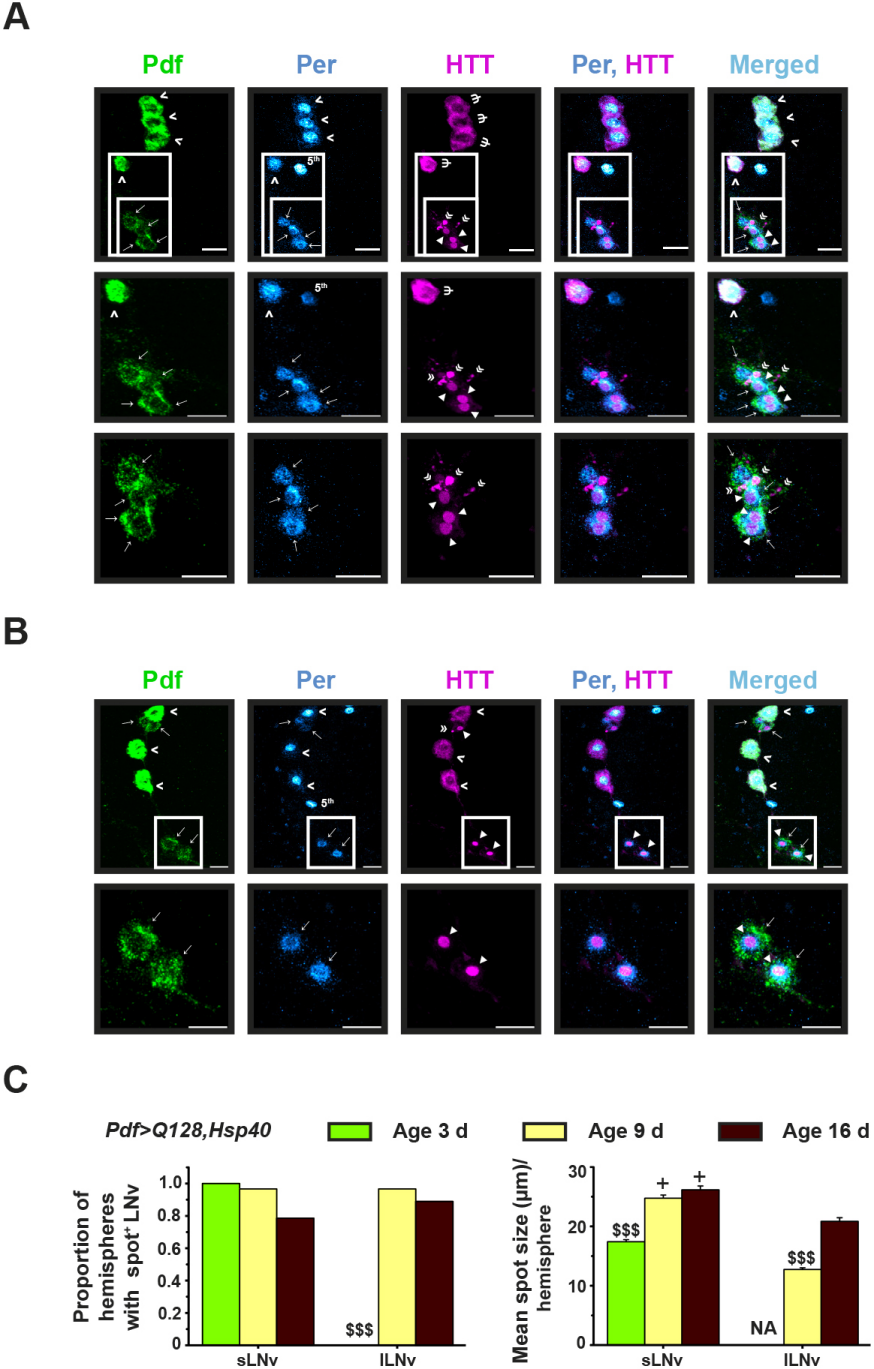
***Pdf>Q128* flies overexpressing Hsp40 show the presence of a novel expHTT form, the ‘Spot’.** (A,B) Two sets of representative images of 3-day-old adult brains of *Pdf>Q128,Hsp40* stained for Pdf (green), Per (cyan) and expHTT (magenta), showing better resolved expHTT spots, where marked rectangles in each panel are enlarged in the subsequent panels below. Indicated in the images are sLNv soma (‘→’), lLNv soma (‘>’), diffuse expHTT (‘Ψ’), spot expHTT (‘◄’), expHTT inclusions (‘«’) and the Pdf^−^, Per^+^ fifth sLNv. (C) Across three ages, the proportion of hemispheres with spots in sLNv or lLNv (left) and the mean spot sizes in sLNv and lLNv (right) are compared. ‘$$$’ depicts the difference of one age from other ages at *P*<0.001, and ‘+’ indicates age-matched differences between sLNv and lLNv at *P*<0.0001. NA, not applicable. Error bars are s.e.m. Scale bars: 10 µm. *n* for these analyses are shown in the top-left cell sets of Table S3.

Hsps are molecular chaperones that are known to interfere with various stages of aggregation and modify the nature, conformation and solubility of expHTT inclusions (Barral et al., 2004; Wyttenbach and Arrigo, 2009; Lotz et al., 2010; Arrasate and Finkbeiner, 2012). We used immunocytochemistry and light microscopy to determine whether Hsp overexpression modifies the expHTT forms detected in the LNv of *Pdf>Q128*. As detailed in the Materials and Methods section, the expHTT forms present in each sLNv or lLNv were categorised into different types based on their appearance. Interestingly, *Pdf>Q128* with overexpressed Hsp40 had an additional expHTT form that has not been observed in these flies and instances of which seem unreported in the literature. Visually and qualitatively, this form of HTT-Q128 appeared as a compact oval and was excluded from the cytoplasmic Pdf staining (Fig. 3, middle-right panel sets, see ‘◄’; Fig. 5A,B). We refer to this as the ‘Spot’ form of expHTT. Per at circadian time (CT)23 was mainly nuclear, and this compact Spot expHTT form appeared in the vicinity of nuclear Per and might be peri-nuclear (Fig. 5A,B). The Spot form was also restricted to a single structure per LNv. Further, the spots appeared to be present throughout the circadian cycle, and, when Per oscillations were examined, a similar number of expHTT spots was observed at both CT23 and CT11.

Only the *Pdf>Q128,Hsp40* showed the presence of expHTT spots (Fig. 3). Within each hemisphere, Spot expHTT was present in ∼75% of sLNv (three of four) at 3 days and ∼50% of sLNv (two of four) at 9 days and 16 days (Fig. S3A, top). At 3 days and 9 days, nearly every hemisphere of *Pdf>Q128, Hsp40* had at least one sLNv with Spot expHTT, which decreased to∼75% at 16 days (Fig. 5C, left). In lLNv of *Pdf>Q128,Hsp40*, Spot expHTT was absent at 3 days, detected in ∼60% lLNv per hemisphere (two to three of four) at 9 days as Spot accompanied by uniform diffuse expHTT staining (Diff+Spot) and in ∼50% lLNv at 16 days as a distinct Spot (Fig. S3A, bottom). Across samples of *Pdf>Q128,Hsp40*, most of the hemispheres showed the presence of Spot in at least one lLNv (as Diff+Spot or Spot) at 9 days and 16 days (Fig. 5C, left). On average, sLNv of older flies had significantly bigger spots (∼25 µm) than those of 3 days flies (∼17 µm), and in lLNv, a similar trend was seen, with larger spots at 16 days (∼20 µm) than at 9 days (∼12 µm) (Fig. 5C right). The spots in the sLNv were larger than those in age-matched lLNv (Fig. 5C, right).

**Fig. 6.**
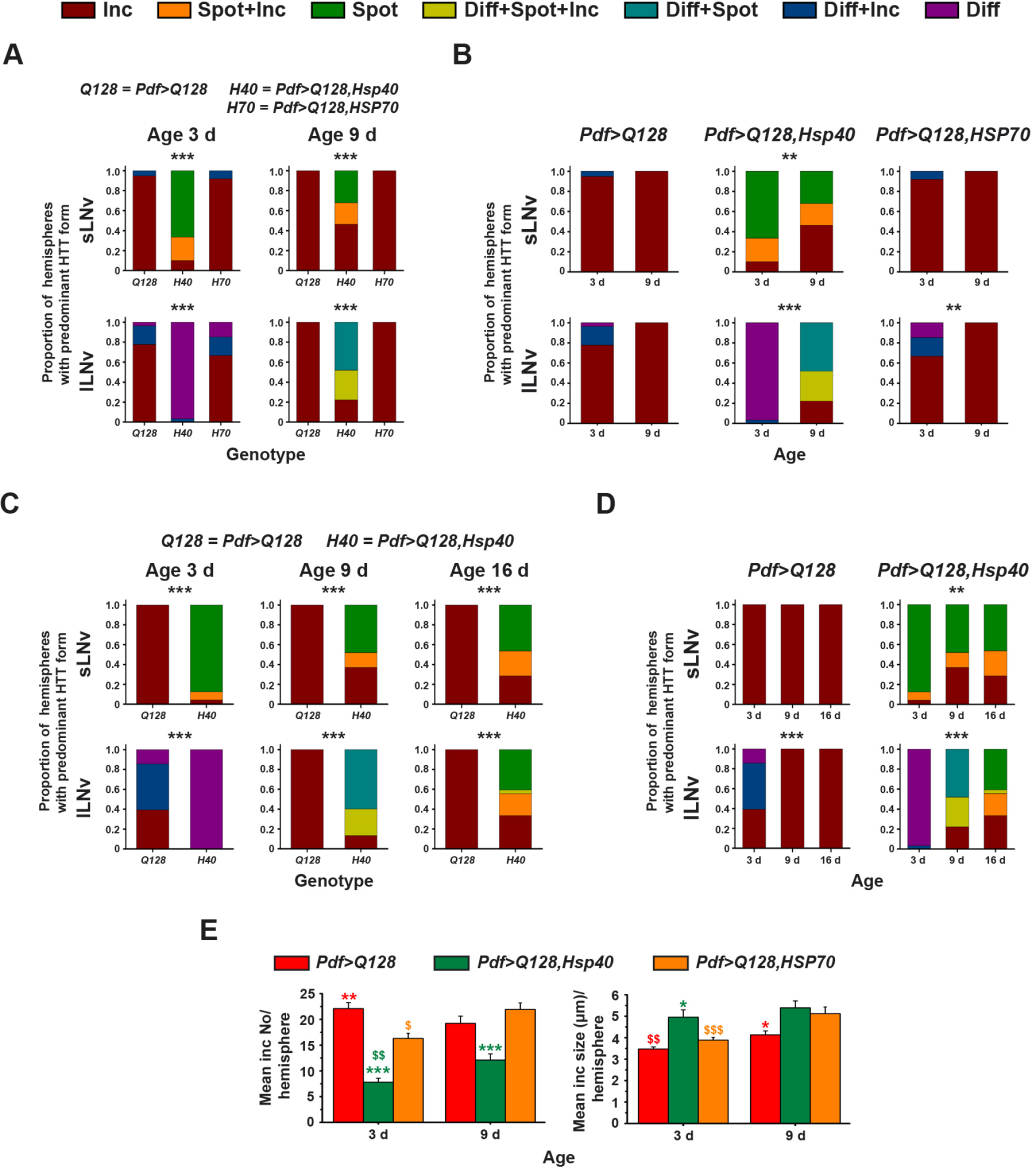
***Pdf>Q128* flies overexpressing Hsp40 have fewer hemispheres with expHTT inclusion-enriched LNv and reduced expHTT inclusion numbers.** (A,B) The proportion of hemispheres dominated by different expHTT forms in sLNv (top) or lLNv (bottom) is plotted on the *y*-axis to describe the between-hemispheres (inter-hemisphere) distribution of predominant expHTT forms. This proportion is plotted at 3 days and 9 days against three genotypes (A), or for each genotype against ages 3 days and 9 days (B). Significant changes in relative distributions of expHTT forms between genotypes (A) or between ages (B) are indicated at **P<0.01 and ***P<0.0001. The relevant pairwise comparisons of A are plotted in Fig. S3B,C. (C,D) Similar to A and B, comparing the three ages, 3 days, 9 days and 16 days, for *Pdf>Q128* and *Pdf>Q128,Hsp40*. The relevant pairwise comparisons of D are plotted in Fig. S4A-C. (E) Comparison of mean inclusion number per hemisphere (left) and mean inclusion size per hemisphere (right) for three genotypes at 3 days and 9 days. Coloured ‘*’ indicates statistically significant age-matched differences between genotypes: green ‘*’ from *Pdf>Q128,Hsp40* and red ‘*’ from *Pdf>Q128*. Coloured ‘$’ represents differences across age for the respective-coloured genotype. Statistical significance represented by symbols: single, *P*<0.05; double, *P*<0.01; triple, *P*<0.001. Error bars are s.e.m. *n* for analyses in A, B and E are shown in the bottom cell sets of Table S3. *n* for analyses in C and D are shown in the top-left cell sets of Table S3.

Comparing the between-hemisphere distribution of predominant expHTT forms in LNv, it is apparent that expHTT appearing as puncta-like shiny specks of varying sizes, referred to as inclusions (Inc), dominated at 3 days and 9 days in both the sLNv and lLNv of *Pdf>Q128* and *Pdf>Q128,HSP70* (Fig. 3, Fig. 6A,B). In contrast, in the LNv of *Pdf>Q128,Hsp40*, non-inclusion forms of expHTT like Diff and Spot dominated over exclusively Inc (Fig. 6A,B). At both ages, the overall distributions of expHTT forms in sLNv and lLNv of *Pdf>Q128,Hsp40* differed significantly from those of *Pdf>Q128* and *Pdf>Q128,HSP70* (Fig. 6A). We then compared the relative proportion of hemispheres in various pairwise category combinations. Specifically, at 3 days, the proportion of hemispheres dominated by Inc expHTT in sLNv relative to Spot forms was significantly higher in *Pdf>Q128* and *Pdf>Q128,HSP70* than that in *Pdf>Q128,Hsp40*, which mostly had hemispheres dominated by Spot expHTT and to a lesser extent Spot+Inc in the sLNv (Fig. S3B, top). At 9 days, nearly 50% of *Pdf>Q128,Hsp40* hemispheres still had Spot-enriched sLNv either as Spot or Spot+Inc expHTT, while a similar proportion of hemispheres also had Inc-enriched sLNv (Fig. S3B, bottom). It is of note that, by 9 days, more than 50% of *Pdf>Q128* and *Pdf>Q128,HSP70* had no Pdf^+^ sLNv, with a mean number of approximately one, whereas nearly all *Pdf>Q128,Hsp40* had four Pdf^+^ sLNv (Fig. S3B, mean Pdf^+^ LNv numbers are indicated at the bottom of each bar).

The proportion of hemispheres with Inc-enriched lLNv relative to other forms of expHTT was significantly higher in *Pdf>Q128* and *Pdf>Q128,HSP70* than *Pdf>Q128,Hsp40* at both 3 days and 9 days (Fig. S3C). *Pdf>Q128,Hsp40* at these ages mostly favoured hemispheres dominated by lLNv enriched with non-Inc forms. At 3 days, most of the hemispheres of *Pdf>Q128,Hsp40* had Diff expHTT in lLNv, and, by 9 days, Spot expHTT appeared, giving rise to hemispheres predominated by mostly non-Inc expHTT forms in lLNv, namely Diff+Spot and Diff+Spot+Inc (Fig. S3C). Thus, the inclusion form of expHTT predominates over other forms in LNv across age in *Pdf>Q128* and *Pdf>Q128,HSP70*, whereas in *Pdf>Q128,Hsp40*, diffuse and spot forms are prevalent.

To track the progress of these distinct forms of expHTT over a more extended duration in the presence of Hsp40, in a separate experiment with *Pdf>Q128* and *Pdf>Q128,Hsp40*, we quantified cellular phenotypes up to 16 days. Like the previous experimental results (Fig. 6A,B), across age, the relative proportions of hemispheres with different expHTT forms in sLNv and lLNv differed significantly between genotypes (Fig. 6C,D). Most of the *Pdf>Q128* hemispheres had Inc-enriched sLNv across age and Inc-enriched lLNv at 9 days and 16 days; in *Pdf>Q128,Hsp40*, Spot expHTT in various combinations dominated the LNv (Fig. 6C,D). In *Pdf>Q128*, as the flies aged, there was a significant reduction in the proportion of hemispheres predominating in Diff or diffuse expHTT with a few puncta-like inclusions (Diff+Inc) expHTT in lLNv relative to those predominating in Inc expHTT (Fig. S4A). In hemispheres of *Pdf>Q128,Hsp40*, Spot-enriched sLNv were present across age (Fig. S4B), and, with age, the proportion of hemispheres with predominantly Spot-enriched sLNv relative to Inc-enriched diminished (Fig. S4B, left). *Pdf>Q128,Hsp40* showed a significant change across age in the proportion of hemispheres dominated by Diff-enriched lLNv relative to those dominated by lLNv enriched by other expHTT forms (Fig. S4C, top row). Post-3 days, Diff expHTT steeply declined in lLNv, making way for Diff+Spot, Diff+Spot+Inc and Inc at 9 days and Spot, Spot+Inc and Inc at 16 days (Fig. S4C, top row). From 9 days to 16 days, the relative proportions of hemispheres of Diff+Spot- (and Diff+Spot+Inc)-enriched lLNv to that of Inc-enriched lLNv decreased significantly (Fig. S4C, middle row, first and second panels). Concomitantly, the relative proportions of hemispheres of Spot (and Spot+Inc)-enriched lLNv to that of Inc-enriched lLNv increased significantly (Fig. S4C, middle row, third and fourth panels). Thus, the overexpression of Hsp40 in *Pdf>Q128* flies decreases the expHTT inclusions in LNv, in favour of expHTT spots. HSP70 overexpression, on the other hand, did not decrease expHTT inclusions in LNv.

In summary, Hsp40 overexpression improves LNv health by reducing inclusions of expHTT in favour of a new form of expHTT, the ‘Spot’, and preserving Pdf^+^ sLNv. The Spot expHTT might be a relatively less toxic form of expHTT, given the control-like Pdf^+^ sLNv numbers of *Pdf>Q128,Hsp40*. Also, *Pdf>Q128,HSP70* and *Pdf>Q128* were nearly indistinguishable in the dominance of expHTT inclusions in LNv, suggesting that the mechanisms mediating early-age rhythm restoration upon HSP70 overexpression might not involve mitigation of visible inclusions.

### Hsp40 overexpression in *Pdf>Q128* flies reduces the number of expHTT inclusions

We quantified the number and size of expHTT inclusions found in and around the LNv. At both 3 days and 9 days, *Pdf>Q128,Hsp40* had significantly fewer inclusions than *Pdf>Q128* and *Pdf>Q128,HSP70* (Fig. 6E, left). At 3 days, *Pdf>Q128,HSP70* also had fewer inclusions than *Pdf>Q128*, but not at 9 days. Both *Pdf>Q128,Hsp40* and *Pdf>Q128,HSP70* exhibited an increase in inclusion number with age. Altogether, these results indicate that overexpression of Hsp40 or HSP70 in *Pdf>Q128* reduces expHTT inclusion numbers, with Hsp40 having lasting effects.

The mean inclusion size of *Pdf>Q128,Hsp40* was higher than that of *Pdf>Q128* and *Pdf>Q128,HSP70* at 3 days, which is likely a reflection of including the relatively large-sized expHTT spots among inclusions during quantification (Fig. 6E, right). Surprisingly, at 9 days, *Pdf>Q128* had smaller inclusions than those of *Pdf>Q128,Hsp40* and *Pdf>Q128,HSP70*. Inclusion size of *Pdf>Q128* and *Pdf>Q128,HSP70* increased with age.

In summary, co-expression of expHTT with Hsp40 in the LNv decreases the proportion of hemispheres with inclusion-enriched LNv across age, with a concomitant increase in the proportion of hemispheres enriched in a hitherto unreported Spot form of expHTT in LNv and a decrease in the expHTT inclusion numbers. All the above observations, taken together, will be henceforth referred to as a decrease in the ‘inclusion load’. Thus, Hsp40 overexpression in *Pdf>Q128* flies reduces the inclusion load of the LNv.

### Hsp40 rescues early-age sLNv Per oscillations in expHTT-expressing flies

**Fig. 7.**
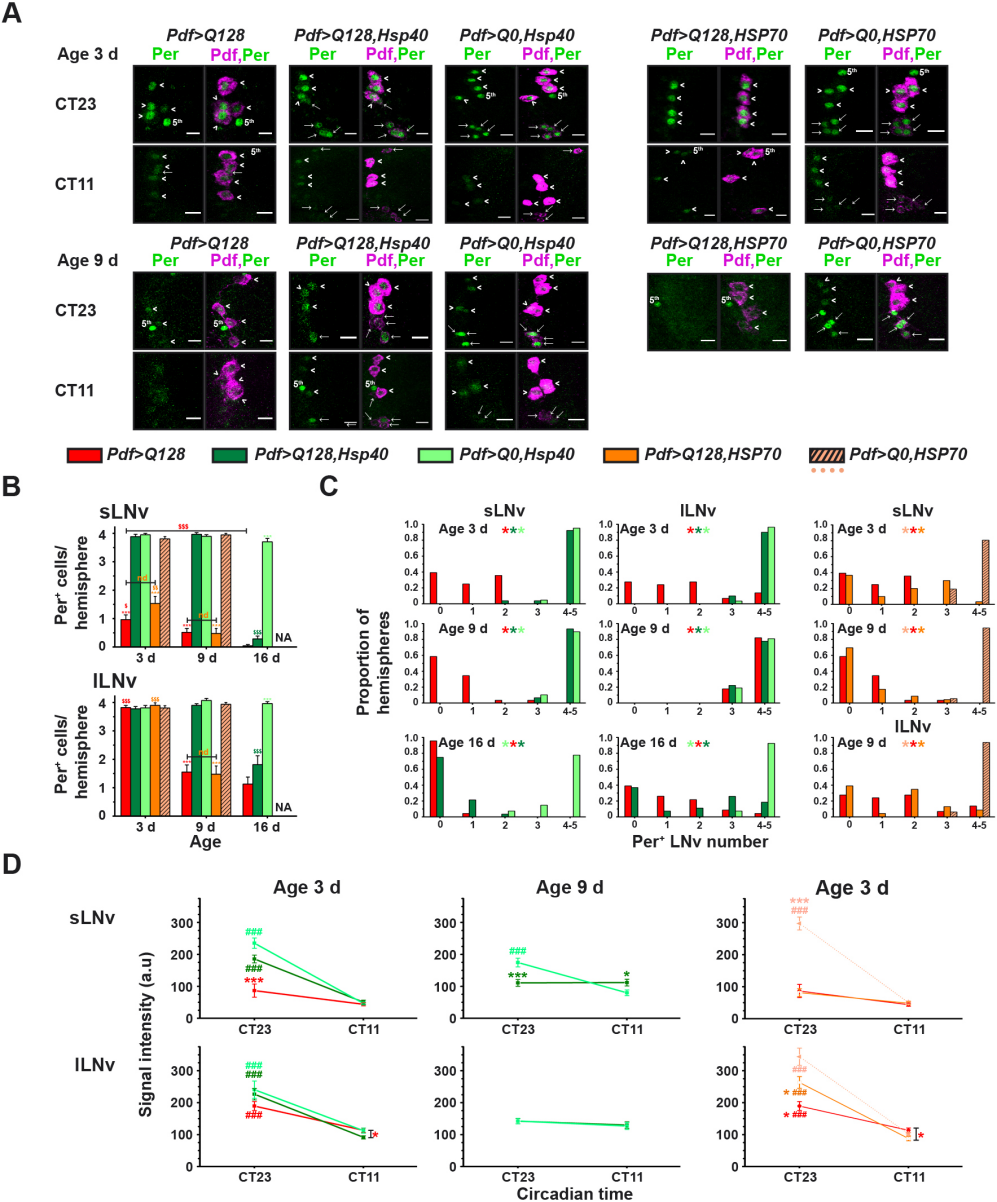
**Young *Pdf>Q128* flies co-expressing Hsp40 show Per oscillations in sLNv.** (A) Representative images of adult fly brains stained for Per (green) and Pdf (magenta) in LNv at circadian time (CT)23 and CT11. sLNv soma (‘→’), lLNv soma (‘>’) and Pdf^−^ Per^+^ fifth sLNv are indicated. Top panel sets: 3-day-old flies of five genotypes. Bottom panel sets: first three columns are of 9-day-old flies of *Pdf>Q128*, *Pdf>Q128,Hsp40* and *Pdf>Q0,Hsp40* at CT23 and CT11; fourth and fifth columns are of 9-day-old *Pdf>Q128,HSP70* and *Pdf>Q0,HSP70* at CT23. Scale bars: 10 µm. (B) Mean number of Per^+^ sLNv soma (top) and lLNv soma (bottom) at three ages at CT23. Symbols indicate significant differences, ‘*’ for age-matched, inter-genotype differences and ‘$’ for differences between age for each genotype: red ‘*’ for *Pdf>Q128* from all other genotypes except *Pdf>Q128,HSP70*, and orange ‘*’ for *Pdf>Q128,HSP70* from other genotypes except *Pdf>Q128*. nd, not different. NA, not applicable. (C) Frequency distribution of the proportion of hemispheres having 0-5 Per^+^ LNv soma: sLNv soma (left column) and lLNv soma (middle column) in *Pdf>Q128,Hsp40*, *Pdf>Q128* and *Pdf>Q0,Hsp40* at 3 days, 9 days and 16 days; sLNv soma at 3 days (right column, top) and 9 days (right column, middle) and lLNv soma at 9 days (right column, bottom) in *Pdf>Q128,HSP70*, *Pdf>Q128* and *Pdf>Q0,HSP70*. a.u., arbitrary units. Multiple coloured ‘*’ indicates significantly differing shapes of distribution between genotypes, with the first colour of the reference genotype and the subsequent colours of genotypes differing from the reference at *P*<0.01. (D) Quantification of Per intensity at CT23 and CT11 in sLNv (top row) and lLNv (bottom row), comparing *Pdf>Q128* with *Pdf>Q128,Hsp40* and *Pdf>Q0,Hsp40* at 3 days (left column) and 9 days (middle column), and comparing *Pdf>Q128* with *Pdf>Q128,HSP70* and *Pdf>Q0,HSP70* at 3 days (right column). Differences between time points CT23 and CT11 are represented by red ‘#’, *Pdf>Q128*; dark-green ‘#’, *Pdf>Q128,Hsp40*; light-green ‘#’, *Pdf>Q0,Hsp40*; dark-orange ‘#’, *Pdf>Q128,HSP70*; and light-orange ‘#’, *Pdf>Q0,HSP70*. Coloured ‘*’ represents age-matched differences of respective-coloured genotype from the indicated one or all others at that timepoint. Statistical significance represented by symbols: single, *P*<0.05; double, *P*<0.01; triple, *P*<0.001. Error bars are s.e.m. *n* for analyses are shown in Table S3: top-left cell sets i.e. CT23 for B and C, and top cell sets for D.

Per, a central clock protein, is lost from the soma of LNv in *Pdf>Q128* flies (Prakash et al., 2017), also seen here, with *Pdf>Q128* having significantly fewer Per^+^ sLNv soma at 3 days and 9 days and almost none at 16 days (Fig. 7A, leftmost column, B, top; Fig. S4D, left). We addressed whether the neuroprotective effect of Hsp40 overexpression on *Pdf>Q128* flies extends to loss of Per and its molecular oscillations in the LNv. *Pdf>Q128,Hsp40* showed the presence of Per^+^ sLNv and lLNv soma at 3 days and 9 days, with control-like numbers (Fig. 7A, left panel sets, B) and their frequency distributions were left-skewed like for *Pdf>Q0,Hsp40* and differed from *Pdf>Q128* (Fig. 7C, left and middle columns). However, unlike the rescue of Pdf in sLNv soma, Per rescue in the LNv soma was not sustained up to 16 days, by which time *Pdf>Q128,Hsp40* had significantly fewer Per^+^ sLNv and lLNv than those of control and was comparable to *Pdf>Q128* (Fig. 7B; Fig. S4D). The shape of the frequency distribution of Per^+^ sLNv and lLNv soma numbers in *Pdf>Q128,Hsp40* changed from a control-like left-skew at 9 days to a *Pdf>Q128*-like shape at 16 days (Fig. 7C, left and middle panel sets). *Pdf>Q128,HSP70* did not show rescue of Per^+^ sLNv soma across age. Its mean numbers and distribution shapes were comparable to those of *Pdf>Q128* and significantly differed from those of *Pdf>Q0,HSP70* and *Pdf>Q128,Hsp40* (Fig. 7A, right panel sets, B, top, C, right panel sets). Per^+^ lLNv soma numbers of *Pdf>Q128,HSP70* were comparable to those of *Pdf>Q128* at 3 days and 9 days, control-like at 3 days and significantly reduced at 9 days (Fig. 7B, bottom). The shape of the Per^+^ lLNv soma distribution of 9-day-old *Pdf>Q128,HSP70*, like that of *Pdf>Q128*, differed from the left-skewed distribution of *Pdf>Q0,HSP70* (Fig. 7C, bottom right).

Because *Pdf>Q128,Hsp40* showed control-like Per^+^ sLNv soma numbers at 3 days and 9 days, Per oscillations in LNv were assessed at these ages. At 3 days, *Pdf>Q128* did not show Per oscillations in sLNv; *Pdf>Q128,Hsp40* showed a significant oscillation in Per levels like *Pdf>Q0,Hsp40* (Fig. 7A, top-left panel sets, D, top left). The Per intensity in sLNv of *Pdf>Q128* was significantly lower than for the other two genotypes at CT23. However, at 9 days, despite having control-like sLNv numbers and Per in the sLNv, Per oscillation was absent in sLNv of *Pdf>Q128,Hsp40*, with intensity at CT23 being significantly diminished compared to that of *Pdf>Q0,Hsp40* (Fig. 7A, bottom-left panel sets, D, top middle). At 3 days, Per oscillation was seen in lLNv of *Pdf>Q128*, *Pdf>Q128,Hsp40* and *Pdf>Q0,Hsp40* (Fig. 7A, top-left panel sets, D, bottom left). At 9 days, Per oscillations were absent from lLNv of both *Pdf>Q128,Hsp40* and control *Pdf>Q0,Hsp40* (Fig. 7A, bottom-left panel sets, D, bottom middle), as is reported for wild-type flies (Shafer et al., 2002; Veleri et al., 2003). Per oscillations in LNv of *Pdf>Q128,HSP70* were like *Pdf>Q128*, with no Per oscillation in sLNv at 3 days and a significant oscillation in lLNv (Fig. 7A, top-right panel sets, D, right column). HSP70 overexpression did not rescue Per in sLNv, even in young *Pdf>Q128* flies. Thus, in young expHTT-expressing flies, Hsp40 overexpression restores both Per numbers and Per oscillations in the sLNv. This study is the first thus far to report rescue in circadian molecular oscillations accompanying the restoration of behavioural rhythms observed in these flies, underscoring the effectiveness of Hsp40 as a potent circadian modifier in HD.

Hsp40 overexpression in LNv of expHTT flies leads to the rescue of sLNv circadian clock output, molecular oscillations and their associated behavioural rhythms in young flies. The rescue of behavioural rhythms and the clock output Pdf is long-lasting. There is also a considerable reduction in the expHTT inclusion load. This sustained rescue at multiple levels posits Hsp40 as a potential therapeutic candidate for improving circadian health under neurodegenerative conditions.

## DISCUSSION

### Hsps as modifiers of HD-induced circadian dysfunction

The role of Hsps, known modifiers of neurodegeneration, in HD-associated circadian disturbances is relatively unexplored. In this study, we show that the overexpression of the co-chaperone Hsp40 in circadian pacemaker neurons of *Drosophila* delays expHTT-induced circadian behavioural arrhythmicity over extended durations and suppresses circadian neurotoxicity. Overexpression of the central chaperone HSP70 also mitigated expHTT-induced circadian behavioural rhythm disruptions in young flies but did not rescue cellular phenotypes. The rescue upon Hsp40 overexpression was more robust, pronounced and sustained. In young flies, Hsp40 overexpression seemed to restore the functionality of LNv, particularly the central pacemaker sLNv. Evidence for sLNv functionality is the restoration of circadian proteins, the core-clock protein Per, its oscillations and the output neuropeptide Pdf in the sLNv soma, and lowered expHTT inclusion load, leading to an overall improvement in the LNv circuit-associated behavioural rhythms. These flies continued to be behaviourally rhythmic up to 16 days but with lowered robustness, with the presence of Per in LNv and control-like Pdf^+^ sLNv soma numbers and diminished inclusion load, but without Per oscillations. The persistence of activity rhythms in the absence of Per oscillations in sLNv suggests two conclusions. First, that Hsp40-mediated rescue at the circadian output level seems sufficient for behavioural rhythm rescue. Second, the Per oscillations in the sLNv might be dispensable for rhythm sustenance. Two recent studies that support this reasoning show that Per in LNv does not seem necessary for the persistence of free-running activity rhythms but is vital for rhythm strength (Delventhal et al., 2019; Schlichting et al., 2019). In relatively older flies, despite the presence of nearly four Pdf^+^ sLNv soma (16 days) and reduction in the inclusion load, the flies were arrhythmic during AW3 (16-23 days). Thus, the Hsp40 neuroprotection does not seem sufficient as the flies age, contributing to a deterioration of LNv health. The inadequacy of Hsp40 expression to extend protection to LNv for extended durations suggests two conclusions. First, the restoration of Pdf^+^ sLNv does not guarantee sustained free-running rhythms in the absence of Per. Our previous results show that ∼20% of 7-day-old arrhythmic *Pdf>Q128* flies had at least one to two Pdf^+^ sLNv. Also, the Pdf levels in the sLNv dorsal projections were oscillating and functional in synchronising downstream circadian neurons (Prakash et al., 2017). The previous results and present observations of behavioural arrhythmicity in older flies despite Pdf rescue in sLNv indicate that sLNv Pdf, in the absence of Per, is insufficient for rhythmic activity. Second, over time, neuroprotective benefits offered by Hsp40 can be overwhelmed upon HD progression, probably by the age-related burden on LNv proteostasis, rendering the cells vulnerable to expHTT toxicity. Therefore, sustained rhythm rescue might require supplementing Hsps with further enhancement of proteostasis via proteasomal or autophagic upregulation.

HSP70 overexpression, in contrast, showed a rescue in only early-age rhythms and activity consolidation, albeit of lowered robustness, a decrease in early-age inclusion number, without the rescue of Pdf^+^ sLNv numbers or Per oscillations in sLNv or alterations to inclusions being the prevalent form of expHTT in LNv. The rhythmic flies of *Pdf>Q128,HSP70* have poor rhythm robustness and can be attributed to the absence of Per rescue in the LNv. However, the persistence of behavioural rhythms with HSP70 overexpression in the absence of Pdf rescue in the sLNv soma is intriguing. It suggests that the presence of Pdf in sLNv is not necessary for behavioural rhythm restoration. Other studies in *Pdf>Q128* have reported rhythm rescue with only marginal Pdf restoration in sLNv soma upon Atx2 or Hop downregulation (Xu et al., 2019b; Xu et al., 2019a). Together with ours, these reports suggest that other mechanisms might drive circadian behavioural rhythms without canonical circadian cellular proteins. Possible intersections of Hsp onto improving LNv function and output in orchestrating rhythmicity are circadian oscillations in arborisations of the sLNv termini and secondary molecular loop components, non-Per driven clocks, LNv membrane properties, neuronal firing, synaptic strength and network-level communication (Edgar et al., 2012; Beckwith and Ceriani, 2015; Yao et al., 2016; Rey et al., 2018; Bulthuis et al., 2019).

Circadian disturbances in HD stem from perturbations to the circadian organisation's input, molecular oscillator and output components (Fifel and Videnovic, 2020; Colwell, 2021). In R6/2 HD mice, free-running activity rhythms are disrupted, and the SCN molecular oscillations are impaired *in vivo*, while persisting in organotypic slices, suggesting that the inputs to and outputs from the central clock are affected rather than the molecular clock (Morton et al., 2005; Pallier et al., 2007). Dysfunctional intrinsically photosensitive retinal ganglion cells, reduction of VIP immunostaining in the SCN, and disrupted rhythms in SCN electrophysiology, cortisol, melatonin, body temperature, heart rate and metabolic outputs (Smarr et al., 2019; Fifel and Videnovic, 2020; Colwell, 2021) provide evidence for circadian disturbances in HD mice at levels of clock input and output, thus also affecting molecular clockwork *in vivo*, resulting in overt behavioural and peripheral rhythm disturbances. Although the HD flies used in this study had a subset of their clock neurons targeted, they exhibited a definite circadian disturbance in the overt behavioural rhythms, molecular oscillations, and circadian output neuropeptide Pdf, recapitulating the central clock and circadian output impairments seen *in vivo* in HD mice. Such parallels between model systems suggest the possibility of finding mammalian counterparts to the Hsp40-mediated rescue of circadian disturbances. Whether the benefits of Hsp40 treatment extend to other circadian rhythms remains to be elucidated.

### Impact of Hsp overexpression on visible inclusions of expHTT

Hsps dilute the presence of aggregate-prone proteins by interfering with the aggregation pathway by delaying nucleation, fibril elongation or redirecting the pathway towards less-toxic versions, sequestrating intermediates into cellular compartments or organelles and targeting for degradation (Kampinga and Bergink, 2016; Mannini and Chiti, 2017; Hipp et al., 2019). The effects of Hsps on aggregation vary, depending on a host of factors such as the definition of aggregates, their nature and conformation, the cellular context, the stage of aggregation, age and disease stage, quantification method and model system. Indeed, with upregulation of Hsps (Hsp40 and Hsp70) in HD models, there is evidence for differential effects: many show a decline in aggregation (Jana, 2000; Zhou et al., 2001; Hay, 2004; Guzhova et al., 2011; Labbadia et al., 2012; Maheshwari et al., 2014; Scior et al., 2018), some show no effect (Wyttenbach et al., 2000; Karpuj et al., 2002; McLear et al., 2008), and one study shows an increase in aggregation (Wyttenbach et al., 2000).

The present study defines the visible puncta-like clumped appearance of HTT-Q128, as detected under a fluorescence light microscope using immunocytochemistry, as an inclusion. This definition excludes detecting many species below the resolution limit and does not distinguish based on solubility and other biochemical features. Hence, the inferences are limited to the size range and gross features of particles detected via an epifluorescence scope. However, this does not take away the validity of the effect of Hsp40 overexpression on expHTT inclusions and its impact on LNv function at the cellular and behavioural stages. In *Pdf>Q128* flies, Hsp40 overexpression in the LNv leads to a reduction in the number of visible expHTT inclusions, a decline in the dominance of inclusion form of expHTT, and the appearance and dominance of expHTT spots. Accompanying them are improvements in LNv pacemaker function, as evidenced by the re-establishment of circadian molecular and behavioural rhythms. Thus, a decreased inclusion load and the dominance of expHTT spots could lead to enhanced functionality of the LNv.

**Fig. 8.**
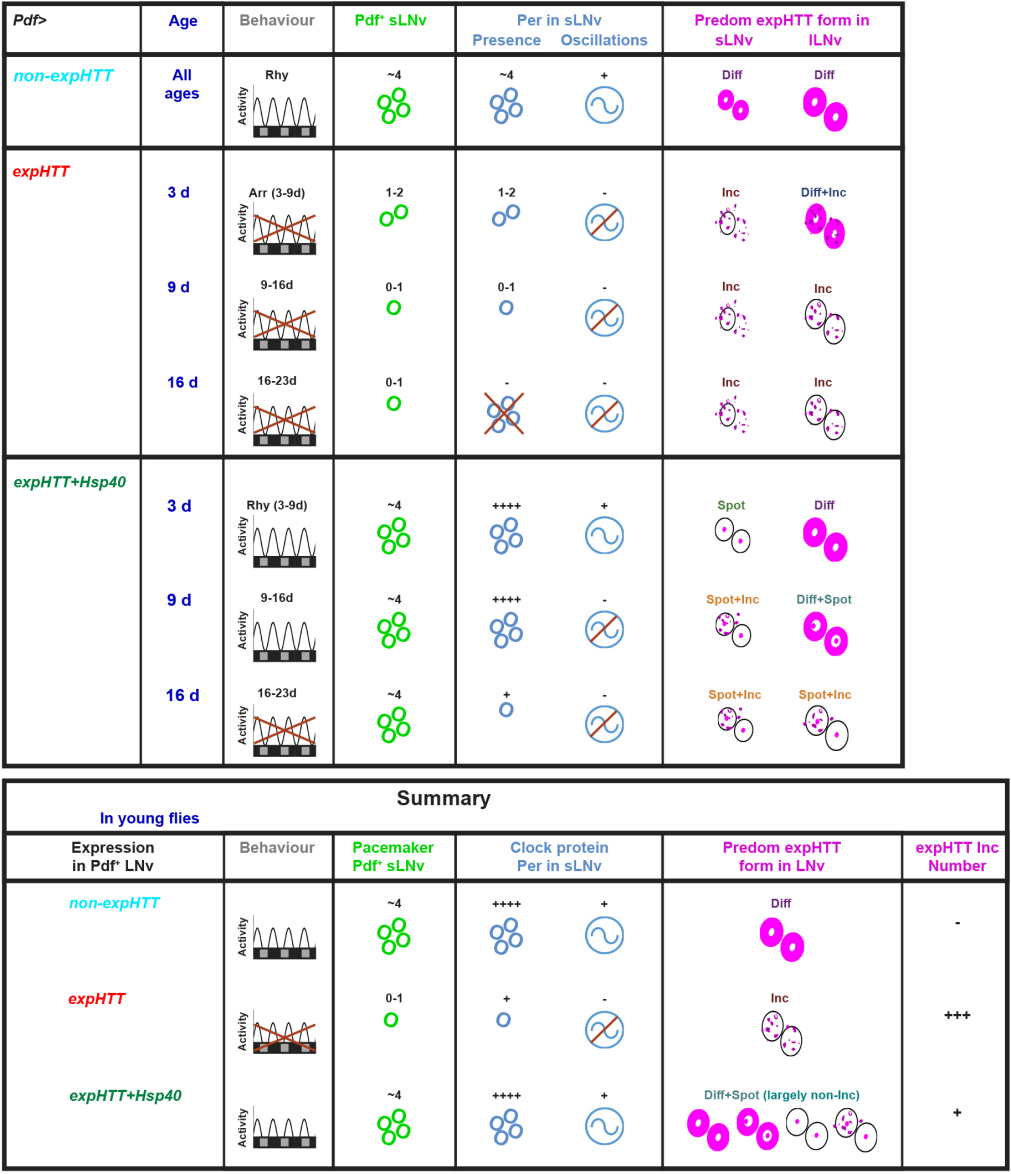
**Hsp40 is neuroprotective and delays circadian dysfunction in Huntington's disease (HD): a graphical summary.** Top table: a pictorial representation of the effect of expressing expHTT alone and with Hsp40 in the LNv of *Drosophila* on circadian neurodegenerative phenotypes across age. The control phenotype expressing non-expHTT (Q0) in LNv is shown at the top. The effects on the circadian behavioural activity/rest rhythms, Pdf^+^ and Per^+^ sLNv soma numbers, Per oscillations in sLNv, and the predominant form of expHTT in sLNv and lLNv are shown across age. The behavioural rhythms are represented for three 7 days age windows, whereas the cellular phenotypes are for specific ages. Arr, arrhythmic; Rhy, rhythmic. Bottom table: a summary of the key findings. Co-expressing Hsp40 with expHTT in the LNv reverses the expHTT-induced circadian phenotypes of behavioural arrhythmicity, Pdf loss from sLNv soma and loss of Per oscillations and Per in sLNv of young flies. Also, the expHTT inclusions, a characteristic neurodegenerative feature, and the predominant expHTT form observed in the LNv of *Pdf>Q128* flies are replaced by mainly non-inclusion forms: diffuse, spots and a combination. The prevalence of non-Inc expHTT forms is also reflected as a decrease in the number of expHTT inclusions. In summary, Hsp40 is an effective suppressor of HD-induced circadian disruptions.

A pictorial representation of the locomotor behaviour and the LNv cellular phenotypes comparing *Pdf>Q128* with *Pdf>Q128,Hsp40* is shown (Fig. 8). A clear pattern for expHTT forms in LNv with age emerges. For the toxic *Pdf>Q128* and relatively less-toxic *Pdf>Q128,HSP70*, Diff+Inc expHTT in lLNv at an early age gives way to Inc only at later ages. In the neuroprotective *Pdf>Q128,Hsp40*, across ages, Spots are present in the sLNv, dominating over Inc at 3 days, whereas Inc dominates at later ages. In the lLNv of *Pdf>Q128,Hsp40*, Diff expHTT dominates at 3 days, giving way to combinations of diffuse, spot and inclusion at 9 days, and then to non-diffuse expHTT (Spot and Spot+Inc) at 16 days. In *Pdf>Q128,Hsp40*, the continued presence and domination of Spot expHTT in LNv is associated with intact Pdf^+^ sLNv and behavioural rhythmicity of most *Pdf>Q128,Hsp40* flies up to 16 days. Together, these results indicate an association between specific expHTT forms predominating in LNv and LNv health, namely diffuse and spot forms with healthy LNv and rhythmic activity, and inclusions with poor LNv function and arrhythmicity.

HSP70 overexpression in *Pdf>Q128* flies rescues early-age rhythms and reduces expHTT inclusion numbers, but with the dominance of inclusion as the main expHTT form in LNv, suggesting that HSP70-mediated improvements to LNv health are via inclusion-independent mechanisms. HSP70 serves aggregation-independent neuroprotective roles such as inhibiting apoptosis (Beere, 2004; Kennedy et al., 2014), combating inflammation (Borges et al., 2012; Dukay et al., 2019), reducing reactive oxygen species and oxidative stress (Kalmar and Greensmith, 2009; Wyttenbach and Arrigo, 2009), and supporting synaptic function (Deane and Brown, 2016; Gorenberg and Chandra, 2017). Studies supporting such aggregation-independent neuroprotection by Hsp40 and Hsp70 in HD are reported (Zhou et al., 2001; Wyttenbach, 2002; Borrell-Pages et al., 2006; Wacker et al., 2009). HSP70 versatility in enhancing neuronal health and function can be attributed to the circadian rhythm improvements in the absence of an effect on the inclusion load and Pdf restoration in the sLNv.

### Spot form of expHTT

Upon overexpression of Hsp40 in *Pdf>Q128* flies, a new form of expHTT with a spot-like appearance located close to the nucleus and seemingly overlapping with the nuclear Per was observed. We discuss the possible significance of the Spot expHTT. In eukaryotes, aggregate-prone proteins are often sequestered into specialised cellular compartments that are thought to be neuroprotective and are typically membraneless, sometimes referred to as sequestrosomes, or into membrane-bound organelles (Sontag et al., 2014; Tan and Wong, 2017; Johnston and Samant, 2021). A few examples of such spatially sequestered quality control sites are the cytoplasmic Q-bodies or stress foci, cytoplasmic p62 bodies or aggresome-like induced structures, peri-nuclear juxta-nuclear quality control compartment, intra-nuclear quality control compartment, peri-nuclear aggresomes and peri-vacuolar insoluble protein deposit (Tan and Wong, 2017; Johnston and Samant, 2021). Hsps are known to participate in such compartmentalisations (Nollen et al., 2001; Specht et al., 2011; Escusa-Toret et al., 2013; Miller et al., 2015). The aggresomes are mostly juxta-nuclear, membrane-free inclusions carrying ubiquitinated misfolded proteins formed at the microtubule organising centre (Johnston et al., 1998; Kopito, 2000; Johnston and Samant, 2021). There is evidence for colocalisation of Hsp40 and Hsp70 with aggresomes (García-Mata et al., 1999; Junn et al., 2002; Gamerdinger et al., 2011; Zhang and Qian, 2011).

Another observation here is that the average size of an expHTT Spot in the small LNv is significantly larger than those in the large LNv. This sizeable expHTT Spot in the sLNv could reflect a greater expHTT burden and toxicity in the vulnerable sLNv or a longer duration of expression of HTT and Hsp40 owing to their earlier appearance than the lLNv during development (Helfrich-Forster, 1997). The presence of three to four Pdf^+^ sLNv in nearly every hemisphere of *Pdf>Q128,Hsp40* across age parallels with the presence of at least one sLNv per hemisphere having Spot expHTT (proportion of hemispheres with at least one Spot^+^ sLNv: 3 days, 100%; 9 days, ∼97%; 16 days, ∼79%). Such co-occurrences indicate that the appearance of expHTT spots might be protective. Thus, Hsp40 might modify the nature of expHTT inclusions, and the spots could represent a less reactive and relatively benign form of expHTT, contributing to an enhancement in LNv health and function.

### Effect of Hsp40 and HSP70 co-expression

Many studies in PolyQ models have described a synergistic effect upon co-expression of Hsp40 and Hsp70 on the toxicity: expression of both offered better protection than either alone (Chan et al., 2000; Jana, 2000; Kobayashi et al., 2000; Muchowski et al., 2000; Sittler et al., 2001; Bailey et al., 2002; Bonini, 2002; Rujano et al., 2007). The current study shows a synergistic improvement in young HD flies' circadian rhythms in daily activity consolidation, but not in percentage rhythmicity or robustness. The synergistic effect seems subtle, evident with a daily readout like ‘*r*’, but not with a 7 days overt readout of rhythmicity. Over time, there was a decline in rhythm robustness of *Pdf>Q128* flies expressing both Hsps than those expressing Hsp40 alone, suggesting that co-expression of multiple Hsps could become detrimental over time. In another study, such co-expression eliminated the survival benefit of HSP70-only expression, enhancing cell death (Ormsby et al., 2013). Some of the drawbacks of Hsp co-expression and Hsp overexpression, like their pro-carcinogenic effect and generation of seeding-competent nuclei, call for caution when targeting central Hsps for therapy (Jäättelä, 1995; Nylandsted et al., 2002; Tittelmeier et al., 2020b).

### Hsp40 versus HSP70: Hsp40, a superior suppressor of HD neurotoxicity?

In our study, Hsp40 emerges as a superior suppressor of most expHTT-induced phenotypes examined and in terms of the duration of rescue. There is substantial support for Hsp40 being a more effective HD neurotoxic modifier. Among the HSP40, HSP70 and HSP110 chaperone families, the DNAJB class of HSP40 emerged as the most potent protector against PolyQ toxicity (Hageman et al., 2010), and, in R6/2 mice, Hsp70 suppressed HD only moderately (Hansson et al., 2003; Hay, 2004; Popiel et al., 2012), while Hsp40 members had better success (Labbadia et al., 2012; Kakkar et al., 2016). Hsp40 also prevented the secretion of expanded PolyQ proteins from cultured cells (Popiel et al., 2012), thus likely preventing their cell-to-cell transmission, an emerging concern in NDs. Findings from the present study and other studies (Chai et al., 1999; Zhou et al., 2001; Rujano et al., 2007; Ormsby et al., 2013) show that Hsp40 reduces aggregation more often than Hsp70. Hsp40 is rate limiting in the suppression and reversal of expHTT aggregation by disaggregases (Rujano et al., 2007; Scior et al., 2018), and some members can act without requiring Hsp70 (Hageman et al., 2010; Kuo et al., 2013; Månsson et al., 2013; Kakkar et al., 2016). A study of genetic modifiers of different NDs, including HD in different model organisms, revealed *Drosophila DnaJ-1* (and its mammalian ortholog *Dnajb4*) as a modifier across many NDs (Na et al., 2013). The significant role of the DNAJB protein family in synaptic health and neuronal proteostasis, and their diversity in function, distribution and substrate specificity, underscore their usefulness in directed therapy while minimising the side effects (Chuang et al., 2002; Westhoff et al., 2005; Gibbs et al., 2009; Kampinga and Craig, 2010; Gao et al., 2015; Nillegoda et al., 2018; Kampinga et al., 2019; Tittelmeier et al., 2020a).

### A need for screening circadian-specific neurotoxic modulators

In an *in vivo* system, we have shown a neuroprotective role of chaperone Hsp40 in rescuing HD-induced circadian deficits and neurotoxicity at multiple levels and across the temporal scale. Such a multi-level associated functional rescue offers an edge over conventional non-associated cellular and behavioural rescues such as to make better cause–effect inferences due to reduced off-target effects, rigorously test the robustness and versatility of the modifying treatment, and serve as proof-of-principle evaluations. Also, although most of the candidates screened are known modulators of neurotoxicity, only two groups of proteins emerged as potent suppressors of circadian disruption, uncovering a gap in establishing circadian-specific neuroprotective agents.

### HSPs, circadian health and neurodegenerative diseases

There is ample evidence for clock-controlled regulation of proteostasis components, including chaperones (Desvergne and Friguet, 2017; Ryzhikov et al., 2019; Wang et al., 2020), with both Hsp40 and Hsp70 isoforms showing rhythmic gene expression across taxa (Li et al., 2017). In the *Drosophila* LNv, mRNA transcripts of the Hsp40 isoforms, DnaJ-1 and the DnaJ-H are present, and, in the lLNv, *DnaJ-H* gene expression is cyclic (Kula-Eversole et al., 2010; Ma et al., 2021), while *Hsp70* transcripts cycle in the LNv (Abruzzi et al., 2017; Ma et al., 2021). Further, *Hsp40* gene transcripts have been classified under the experimentally identified circadian genes (Li et al., 2017). The converse, i.e. proteostasis affecting the molecular clock via post-translational modifications and autophagy, is also prevalent (Mehra et al., 2009; Stojkovic et al., 2014; Toledo et al., 2018; Juste et al., 2021). However, very few studies have assessed the role of Hsps in circadian maintenance and its deterioration in NDs, especially in animals. In *Drosophila*, the Hsp70/Hsp90-organising protein (Hop) improved rhythmicity in an HD model (Xu et al., 2019b), Hsp70 expression overcame arrhythmicity due to Gal4-overexpression in the LNv (Rezaval et al., 2007), Hsp90 disruptions led to the loss of activity rhythms without affecting molecular oscillations (Hung et al., 2009), and Hsps were indirectly implicated in circadian behaviour (Benbahouche et al., 2014; Means et al., 2015). In mouse fibroblasts, Hsp90 is required for circadian rhythmicity, while Hsf1 and endoplasmic reticulum Hsp70 strengthen rhythms post-stress (Tamaru et al., 2011; Schneider et al., 2014; Pickard et al., 2019). The present findings of a relatively novel role for the Hsps in protecting against ND-induced circadian dysfunction and the above studies encourage further research on Hsps in circadian function. Given that circadian and sleep disturbances occur early and are pre-manifest in HD (Soneson et al., 2010; Morton et al., 2014; Lazar et al., 2015; Bellosta Diago et al., 2017), treatments targeting HSPs could impact HD's early stages in postponing symptoms and provide a meaningful therapeutic impact. An ageing population worldwide has increased the prevalence of NDs (Gitler et al., 2017; Lassonde et al., 2017; Béjot and Yaffe, 2019). Given the pivotal roles of proteostasis and circadian health in NDs, studying the involvement of molecular chaperones in circadian maintenance will significantly improve our understanding of ND progression and treatment.

## MATERIALS AND METHODS

### Fly lines

The *UAS-HTTpolyQ* lines used in this study, namely *w;+;UAS-HTT-Q0A;+* (without Q repeats) and *w;+;UAS-HTT-Q128C;+* (with 128 polyQ repeats), were a gift from Troy Littleton, Massachusetts Institute of Technology (Lee et al., 2004). They contain the first 548 amino acids of the human *HTT* gene. The *UAS* males were crossed with virgin females of either *w;PdfGal4:+* or *w^1118^:+:+* (BL 5905) to generate fly lines expressing HTT-polyQ in the Pdf^+^ LNv neurons designated *Pdf>Q0* and *Pdf>Q128* or the *UAS* background controls designated *Q0* and *Q128*, respectively. In some instances, a broader circadian driver, the *w;timGal4:UAS-GFP* was used for the screen. For the genetic screen, the fly lines used are listed in Table S1. The genes were either overexpressed or downregulated in the *Pdf>Q128* background (and in a few cases, *tim>Q128* background) (Table S1). A recombinant line of *w;PdfGal4;+* and *w;UAS-HTT-Q128/+;+* was generated, denoted as *w;Pdf-Q128;+*, and used for the screen. The sample size for the screen varied between 16 and 32 flies per genotype.

For most of this study, the fly lines of focus were generated using the two Hsp *UAS* lines, namely *w;+;UAS-DnaJ1k* (Bl 30553) and *w;+;UAS-HSPA1L* (Bl 7054). The human *HSP70* homolog used in this study was *HSPA1L* (Bl 7454) (https://flybase.org/reports/FBgn0029163.html), as a recent analysis revealed that, among the various chaperone families, the HSP70 family most frequently provided considerable proteotoxic relief in a variety of protein misfolding diseases (Brehme and Voisine, 2016). The next potent proteotoxic suppressor was the Hsp40 family, among which DnaJb4 and DnaJb6 were the most potent polyQ disease modifiers (Brehme and Voisine, 2016). The *dHdj1* (*DnaJ1k* or *DnaJ-1* or *Hsp40* or *CG1058*) (Bl 30553) used in this study is a *Drosophila* ortholog of members of the human HSP40 Class B family with varying degrees of homology (*DNAJB5*, *DNAJB4* and *DNAJB1*) (https://flybase.org/reports/FBgn0263106.html).

The *UAS-Hsp* lines were used to generate the *w;PdfGal4/UAS-Q128;UAS-DnaJ1k/+* or *w;PdfGal4/UAS-Q128;HSPA1L/+* lines, which would respectively overexpress Hsp40 or HSP70 in the Pdf^+^ LNv neurons, also expressing HTT-Q128. The above-generated lines are referred to as *Pdf>Q128,Hsp40* and *Pdf>Q128,HSP70* throughout the text. Their *UAS* control lines are referred to as *Q128,Hsp40* and *Q128,HSP70* and their driver control lines as *Pdf>Hsp40* and *Pdf>HSP70*. Their corresponding *Q0* lines are *Pdf>Q0,Hsp40* and *Pdf>Q0,HSP70*. All other relevant background controls were also used. Owing to space constraints, in some figures (Fig. 6A,C; Fig. S3B,C), *Pdf>Q128*, *Pdf>Q128,Hsp40* and *Pdf>Q128,HSP70* are abbreviated as *Q128*, *H40* and *H70*, respectively. Flies co-expressing both Hsp40 and HSP70 along with HTT-Q128 in the LNv are referred to as *Pdf>Q128,Hsp40,70*, and their *Q0* counterparts as *Pdf>Q0,Hsp40,70*. Crosses were maintained under 12 h:12 h light: dark cycles (LD), with ∼200 lux intensity of light phase, at 25°C. Flies were moved to DD 25°C after 2 days post-eclosion for behavioural and immunocytochemical assays. All flies and crosses were maintained on a standard cornmeal medium.

### Behavioural assays

Most of the locomotor activity setup, assay conditions and analyses performed are described in a previous study (Prakash et al., 2017). Briefly, the activity rhythms of 3-day-old virgin male flies were recorded in 7 mm glass tubes using the *Drosophila* Activity Monitoring system 2 (DAM2) from TriKinetics (Waltham, MA, USA). Activity recordings were carried out in an incubator (Percival DR36VL) at 25°C in DD for at least 21 days (3-23 days) with ∼80% humidity. The data obtained were analysed using the Chi-Square periodogram in the CLOCKLAB software (Actimetrics, Wilmette, IL, USA). The periodogram threshold was set at *P*=0.01 (Pfeiffenberger et al., 2010). A fly was considered rhythmic if its periodogram amplitude was above the threshold and confirmed by visual inspection of the actogram by a single-blind analyser. The various activity rhythm features such as rhythmicity, rhythm strength or rhythm robustness and period were calculated over three 7 days windows to view progressive changes with age. The three temporal windows (AWs) spanning 21 days were designated as age window 1 (AW1; 3-9 days), age window 2 (AW2; 10-16 days) and age window 3 (AW3; 17-23 days).

Additionally, to track daily changes in activity/rest rhythms, the extent of activity consolidation ‘*r*’ was also calculated using a MATLAB code as previously described (Prakash et al., 2017) with a few modifications. ‘*r*’ represents the extent to which activity data points are consolidated over a circadian cycle of activity. The activity time series for a genotype was obtained at a resolution of 20 min bins. This time series was divided into cycles of length determined by the period (*T*) of that genotype, thereby identifying each circadian cycle in the time series. Thus, each ‘day’ used to calculate ‘daily’ ‘*r*’ was obtained as a modulo *T* value. On each ‘day’, activity counts were imagined as unit vectors for which direction represented the timepoint (*t*) at which the count occurred within the cycle. Because counts were clustered into 20 min intervals, the data can be represented as vectors with a constant angular separation of 20*2π/*T* radians and magnitudes corresponding to the number of activity counts in each interval. *R* was calculated as the magnitude of the mean of these activity vectors. The rectangular coordinates of the mean vector were obtained (Zar, 2010) using *X*=Σ *A_t._*cosθ*_t_*/Σ *A_t_* and *Y*=Σ *A_t_*sinθ*_t_* / Σ A*_t_*, where *A_t_* represents activity counts at a given timepoint *t* and θ*_t_* represents the vector angle associated with the timepoint. The magnitude of this vector ‘*r*’ was calculated as √*X*^2^+*Y*^2^. The greater the magnitude of ‘*r*’, the better the degree of consolidation, with most activity occurring over a few closely spaced timepoints. The lower the magnitude of ‘*r*’, the poorer the consolidation, with activity spread over time. Given that sometimes period changes were observed across age for a fly, daily cycles were identified separately for each 7 days AW using the mean period values for the corresponding AW. For arrhythmic flies, the cycle length was determined using the mean period of the genotype for that AW. For a fly dying in the middle of an AW, the period of that fly in the prior AW (if any) was used to determine cycle length. The daily ‘*r*’ was averaged across flies to obtain the mean daily ‘*r*’.

At least three independent activity runs were carried out for overexpression of Hsp40 with its *Q0* and *UAS* controls, all giving similar results. For overexpression of HSP70, three independent activity runs were carried out with its *Q0* controls, giving similar results. At least one experiment was carried out with all possible genetic controls for both sets of Hsp40 and HSP70 overexpression experiments. No statistical tests were carried out to determine the minimum required sample sizes. However, as recommended (Kostadinov et al., 2021), one full DAM2 monitor accommodating 32 flies per genotype was set up at the start of the experiment. The average percentage rhythmicity across multiple runs is plotted for Hsp40 and HSP70 overexpression experiments. In addition, the percentage rhythmicity, period, robustness and extent of activity consolidation ‘*r*’ values of a representative run with all relevant controls for each of the above overexpression experiments are plotted. For synergistic effects, a single run was carried out. There were fly deaths in AW3; therefore, AW3 analyses had fewer samples. When the fly numbers went below ten (e.g. a few instances in AW3), those genotypes were excluded from statistical analyses of robustness and period for that AW. Owing to fewer surviving flies across genotypes at later ages in the synergistic effect experiment, the ‘*r*’ value's statistical analyses were restricted to 16 days of age. In cases in which very few flies (*n*<10) were rhythmic, like *Pdf>Q128* number across AWs in the HSP70 overexpression and synergistic effect experiments and during AW3 in Hsp40 overexpression experiments, or *Pdf>Q128,Hsp40* during AW3 in the Hsp40 overexpression experiments or most of the experimental genotypes during AW3 in the synergistic effect experiments, those genotypes were excluded from the between-genotype statistical analyses of period and rhythm robustness for that AW. Indicated in Table S2 are the numbers of surviving flies in AW2 and AW3 across experiments.

### Statistical analysis of activity rhythms

Datasets were first tested for normality using Shapiro–Wilk's test and then for variance homogeneity using Levene's test. Across experiments, the data comparing period or activity consolidation ‘*r*’ between genotypes for an AW or age did not satisfy the ANOVA assumption of normality, despite transformations. So was the case for rhythm robustness in the Hs40 overexpression experiment. Therefore, the non-parametric Kruskal–Wallis test of ranks followed by multiple comparisons of mean ranks was used. For the HSP70 overexpression experiment, datasets comparing rhythm robustness between genotypes for an AW were normally distributed post-transformation, but variances were not always homogenous. Hence, Welch's ANOVA was used, followed by Games-Howell post-hoc test. For the synergistic effect experiment, the data comparing robustness between genotypes for an AW were normally distributed and their variances homogeneous. A one-way ANOVA followed by unequal N HSD tests were carried out. Friedman's test for repeated measures was used to compare robustness, period and ‘*r*’ between AWs or ages for a genotype. Then, Wilcoxon matched-pairs tests (or Conover Test for ‘*r*’) with Bonferroni correction [or Benjamini–Hochberg (BH) procedure to decrease the false discovery rate (FDR) for ‘*r*’; FDR set at 5%] on the pairwise *P*-values were used. A m×n Fisher's exact test, followed by multiple 2×2 Fisher's exact tests with BH procedure on all relevant comparisons, were used (using R) to compare the proportions of rhythmic flies between genotypes for an AW. Cochran Q test on the dichotomous variable rhythmicity (rhythmic and arrhythmic categories) was used to compare the proportion of rhythmic flies between AWs for a genotype, followed by multiple 2×2 McNemar's tests on the dependent samples and Bonferroni correction on pairwise *P*-values. For comparing the mean rhythmicity of multiple independent runs between genotypes or between ages, a repeated-measures ANOVA followed by Tukey's HSD was performed post-arcsine conversion of the square-root-transformed data.

Statistical analyses were executed using STATISTICA^TM^ 7.0 (https://statistica.software.informer.com/7.0/) and R (https://www.r-project.org/). Welch's ANOVA was performed using a Microsoft Excel template from http://www.biostathandbook.com/onewayanova.html (McDonald, 2014), McNemar's test using SciStatCalc (https://scistatcalc.blogspot.com/2013/11/mcnemars-test-calculator.html) and Friedman's test followed by Conover test for ‘*r*’ using ASTATSA (https://astatsa.com/FriedmanTest/).

### Immunocytochemical assays

The dissections and immunocytochemistry procedures performed were as described previously (Prakash et al., 2017). Adult fly brain tissues were dissected in 1× PBS at different ages, fixed with 4% paraformaldehyde at room temperature, blocked and stained with the appropriate primary antibodies, followed by secondary antibodies, and then mounted on slides using 70% glycerol in 1× PBS. Primary antibodies used for double staining were mouse anti-HTT (1:500; MilliporeSigma MAB2166) and rabbit anti-Pdf (1:30,000; Michael Nitabach, Yale University) (Nitabach et al., 2006), and for triple staining, rabbit anti-Per (1:20,000; Jeffrey C Hall, Brandeis University) (Houl et al., 2008), mouse anti-HTT (1:500) and rat anti-Pdf (1:3000) (Jae Park, Vanderbilt University) (Park et al., 2000). Rabbit anti-Per was pre-absorbed onto *per^01^* embryos at 1:100 dilution. Secondary antibodies (1:3000) were from Invitrogen: anti-rabbit Alexa Fluor 488, anti-mouse Alexa Fluor 546, anti-mouse Alexa Fluor 647 and anti-rat Alexa Fluor 555.

For characterising the Per^+^ and Pdf^+^ LNv soma numbers, adult fly brain dissections were carried out in parallel at different ages. Because with *Pdf>Q128,Hsp40*, a sustained rescue for two AWs was seen in behaviour, dissections were carried out on 3-day-old, 9-day-old and 16-day-old flies corresponding to the beginning of AW1, the transition of AW1-AW2 and end of AW2, respectively. With *Pdf>Q128,HSP70*, rhythm rescue was restricted to AW1. Hence, dissections were carried out at 3 days and 9 days, corresponding to the beginning and end of AW1, respectively. For characterising HTT status in LNv, adult fly brain dissections were carried out in parallel for the five genotypes (*Pdf>Q128*, *Pdf>Q0,Hsp40*, *Pdf>Q128,Hsp40*, *Pdf>Q0,HSP70* and *Pdf>Q128,HSP70*) at 3 days and 9 days. Many of the Hsp-expressing flies had periods longer than 24 h. Therefore, the mean period values of the respective genotypes were considered to calculate the CT for dissections to detect Per oscillations in the LNv. For quantifying Per oscillations in DD, LD-reared flies were dissected at CT23-24 (CT23) and CT11-12 (CT11) at different ages: all the five genotypes at 3 days and *Pdf>Q0,Hsp40* and *Pdf>Q128,Hsp40* also at 9 days. These samples were co-stained with anti-Pdf to aid in identifying LNv and anti-HTT. The sample sizes were determined empirically (Table S3). There was no blinding during sample preparation.

### Image acquisition and analysis

The number of Pdf^+^ and Per^+^ LNv and the form of expHTT in the LNv were noted on manual observation of the samples using a Zeiss Axio Observer Z1 epifluorescence microscope with a 63×/oil 1.4 NA objective and without blinding. The collected data were then cross-verified with images captured using a 40×/oil 1.3 NA objective as a *z*-stack of 1 µm intervals. The Pdf-stained sLNv and lLNv were distinguished based on their anatomical location, size and staining pattern. For quantification of Per intensity and expHTT inclusions, the lamp intensity and exposure time were kept constant across samples for an experiment. The Per intensity was calculated from the images described previously in a single-blind manner (Prakash et al., 2017). National Institutes of Health imaging software ImageJ was used for image analysis and quantification (Schneider et al., 2012). For representative images, confocal *z*-stacks were captured using a Zeiss LSM 880, keeping the photomultiplier tube gain gain, offset and laser power below saturating pixels. In the representative images, brightness/contrast adjustments were applied to the whole image for better visualisation of the LNv, especially the sLNv, as they showed less intense and sparser Pdf staining than the lLNv.

### Categorisation and quantification of expHTT forms

The numbers of sLNv and lLNv with different forms of expHTT were noted in each hemisphere. The expHTT forms appearing in each LNv soma were classified based on their appearance under the light microscope as follows: uniform diffuse expHTT staining (Diff), expHTT appearing as puncta-like shiny specks of varying sizes referred to as inclusions (Inc), diffuse expHTT with a few puncta-like inclusions (Diff+Inc) and without expHTT staining (No HTT) (Fig. S1A, top row). With overexpression of Hsp40, we also observed a hitherto unreported form of expHTT, oval with a compact appearance, henceforth referred to as ‘Spot’ expHTT or spot-like expHTT (Spot) (Fig. S1A, bottom row). Also observed less frequently was an LNv with an expHTT spot and the canonical inclusions, giving the spot a distorted appearance. Hence, such forms of expHTT were included under the Inc category. If the spot appeared amidst a diffused expHTT distribution, mostly seen in lLNv of young flies, it was designated as Diff+Spot (Fig. S1A, bottom row). Two sets of information can be gathered from such an exercise of labelling expHTT types per LNv. One is at the level of cells, namely the proportion of sLNv or lLNv with different forms of expHTT within each hemisphere. This comparison (intra-hemisphere) gives an idea of the within-hemisphere variation in LNv expHTT distribution. Our experimental replicates are at the level of hemispheres and not cells, so the within-hemisphere expHTT diversity in cells is only qualitative information. The second set of information is at the level of hemispheres. Each hemisphere was allotted a particular category depending on the most predominant expHTT form found in the sLNv (or lLNv) (Fig. S1B). The categorisation of each hemisphere (inter-hemisphere) based on predominant expHTT form in the LNv (sLNv or lLNv) was as follows: predominantly diffuse distribution (Fig. S1B, top row, left), an equal distribution of diffused and inclusions (Diff+Inc) (Fig. S1B, top row, middle and right), predominantly had Diff+Spot (Fig. S1B, second row, left), predominantly had spots (Spot) (Fig. S1B, second row, middle and right), a near equal mix of diffuse, inclusions and spots (Diff+Spot+Inc) (Fig. S1B, third row, left), an equal distribution of spot and inclusions (Spot+Inc) (Fig. S1B, third row, middle and right), or predominantly had inclusions (Inc) (Fig. S1B, fourth row, middle and right). Hemisphere-level categorisation just described is at the level of experimental replicates. It, therefore, makes room for quantitative statistical analysis and allows for comparisons between genotypes and among ages of the relative proportions of hemispheres enriched in one form of expHTT in sLNv or lLNv versus other forms of expHTT. Upon such a hemisphere-level categorisation, hemispheres in which sLNv or lLNv were without expHTT never dominated, so the ‘No HTT’ category does not exist. The arrangement order used in the figures describing various expHTT forms is based on observations made post-hoc as to the appearance and predominance of various expHTT forms in LNv over time (Fig. S1C). For example, in most *Pdf>Q128* samples, sLNv show Diff expHTT as larvae, and young adults mostly exhibit Diff+Inc in lLNv, followed by Inc as they age. Young *Pdf>Q128,Hsp40* flies mostly show Diff expHTT in their LNv. With age, different combinations of Diff, Spot and some Inc appear and dominate (mostly non-Inc forms), and exclusively Inc becomes more prominent only in much older flies.

### Quantification of expHTT inclusions

The expHTT inclusion number and size were quantified using ImageJ. Maximum-intensity projection images were converted to 8-bit images. Their backgrounds were subtracted (rolling ball radius of 10 pixels), an unsharp mask filter was applied (radius of 1 pixel, mask weight of 0.7) and further processed to sharpen the image and find edges, and then the intermodes threshold was applied. The area in and around the LNv was then marked. The analyse particles tool with size specification of 1 to infinity (in µm) was used to obtain measures of inclusion number and the size of inclusions for each hemisphere. A lower limit of 1 µm was set for size to avoid false positives and background specks. The quantification method did not distinguish between expHTT Inc and Spots, resulting in Spots being included in the inclusion number and size quantifications.

### Statistical analysis of cellular features

For comparing Pdf^+^ or Per^+^ LNv numbers between genotypes or between ages, the Kruskal–Wallis test of ranks, followed by multiple comparisons of mean ranks, was used. The change in the shape of frequency distributions of Pdf^+^ and Per^+^ LNv numbers between genotypes was assessed using Kolmogorov–Smirnov tests with α=0.05, followed by Bonferroni correction on pairwise *P*-values. For comparing inclusion numbers between genotypes or across age, multi-factor ANOVA followed by Tukey's HSD post-hoc test was used on transformed data. Because the variances were not homogenous for inclusion size comparisons, the transformed datasets, primarily normal, were subjected to Welch's ANOVAs followed by Games-Howell post-hoc tests (McDonald, 2014). The datasets were either transformed (where required), to analyse the status of Per oscillations between the timepoints CT23 and CT11 for a genotype or the Per intensities at a timepoint between genotypes, or directly analysed using one-way ANOVA, followed by Tukey's HSD post-hoc test wherever necessary. To compare the proportion of hemispheres predominated by different expHTT forms between genotypes for an age or between ages for a genotype, m×n Fisher's exact tests were used. Wherever necessary, multiple specific 2×2 Fisher's exact test sets with BH procedure on all relevant comparisons were applied (using R). The proportion of hemispheres with Spot^+^ LNv between ages was compared using multiple 2×2 Fisher's exact tests followed by Bonferroni corrections. Spot sizes between ages for sLNv or lLNv were analysed by one-way ANOVA (on transformed data, if required), followed by Tukey's HSD tests. For Spot sizes between sLNv and lLNv, a factorial ANOVA with LNv and age as fixed factors was carried out on transformed data, followed by Tukey's HSD.

OriginPro 8 (https://www.originlab.com/origin), Sigma Plots 11.0 (https://systatsoftware.com/sigmaplot/) and Adobe InDesign 3.0 (https://www.adobe.com/uk/products/indesign.html) were used for making figures.

## Supplementary Material

Supplementary information

## References

[DMM049447C1] Abruzzi, K. C., Zadina, A., Luo, W., Wiyanto, E., Rahman, R., Guo, F., Shafer, O. and Rosbash, M. (2017). RNA-seq analysis of *Drosophila* clock and non-clock neurons reveals neuron-specific cycling and novel candidate neuropeptides. *PLoS Genet.* 13, e1006613. 10.1371/journal.pgen.1006613 28182648 PMC5325595

[DMM049447C2] Arrasate, M. and Finkbeiner, S. (2012). Protein aggregates in Huntington's disease. *Exp. Neurol.* 238, 1-11. 10.1016/j.expneurol.2011.12.013 22200539 PMC3909772

[DMM049447C3] Bailey, C. K., Andriola, I. F., Kampinga, H. H. and Merry, D. E. (2002). Molecular chaperones enhance the degradation of expanded polyglutamine repeat androgen receptor in a cellular model of spinal and bulbar muscular atrophy. *Hum. Mol. Genet.* 11, 515-523. 10.1093/hmg/11.5.515 11875046

[DMM049447C4] Barral, J. M., Broadley, S. A., Schaffar, G. and Hartl, F. U. (2004). Roles of molecular chaperones in protein misfolding diseases. *Semin. Cell Dev. Biol.* 15, 17-29. 10.1016/j.semcdb.2003.12.010 15036203

[DMM049447C5] Bates, G. P., Dorsey, R., Gusella, J. F., Hayden, M. R., Kay, C., Leavitt, B. R., Nance, M., Ross, C. A., Scahill, R. I., Wetzel, R. et al. (2015). Huntington disease. *Nat. Rev. Dis. Primers* 1, 15005. 10.1038/nrdp.2015.5 27188817

[DMM049447C6] Beckwith, E. J. and Ceriani, M. F. (2015). Experimental assessment of the network properties of the *Drosophila* circadian clock. *J. Comp. Neurol.* 523, 982-996. 10.1002/cne.23728 25504089

[DMM049447C7] Beere, H. M. (2004). “The stress of dying”: the role of heat shock proteins in the regulation of apoptosis. *J. Cell Sci.* 117, 2641-2651. 10.1242/jcs.01284 15169835

[DMM049447C8] Béjot, Y. and Yaffe, K. (2019). Ageing population: A neurological challenge. *Neuroepidemiology* 52, 76-77. 10.1159/000495813 30602150

[DMM049447C9] Bellosta Diago, E., Pérez Pérez, J., Santos Lasaosa, S., Viloria Alebesque, A., Martínez Horta, S., Kulisevsky, J. and López Del Val, J. (2017). Circadian rhythm and autonomic dysfunction in presymptomatic and early Huntington's disease. *Parkinsonism Relat. Disord.* 44, 95-100. 10.1016/j.parkreldis.2017.09.013 28935191

[DMM049447C10] Ben-Zvi, A., Miller, E. A. and Morimoto, R. I. (2009). Collapse of proteostasis represents an early molecular event in *Caenorhabditis elegans* aging. *Proc. Natl. Acad. Sci. USA* 106, 14914-14919. 10.1073/pnas.0902882106 19706382 PMC2736453

[DMM049447C11] Benbahouche, N. E. H., Iliopoulos, I., Török, I., Marhold, J., Henri, J., Kajava, A. V., Farkaš, R., Kempf, T., Schnölzer, M., Meyer, P. et al. (2014). *Drosophila* Spag is the homolog of RNA polymerase II-associated protein 3 (RPAP3) and recruits the heat shock proteins 70 and 90 (Hsp70 and Hsp90) during the assembly of cellular machineries. *J. Biol. Chem.* 289, 6236-6247. 10.1074/jbc.M113.499608 24394412 PMC3937688

[DMM049447C12] Bonini, N. M. (2002). Chaperoning brain degeneration. *Proc. Natl. Acad. Sci. USA* 99 Suppl. 4, 16407-16411. 10.1073/pnas.152330499 12149445 PMC139901

[DMM049447C13] Borges, T. J., Wieten, L., van Herwijnen, M. J. C., Broere, F., van der Zee, R., Bonorino, C. and van Eden, W. (2012). The anti-inflammatory mechanisms of Hsp70. *Front Immunol* 3, 95. 10.3389/fimmu.2012.00095 22566973 PMC3343630

[DMM049447C14] Borrell-Pages, M., Canals, J. M., Cordelieres, F. P., Parker, J. A., Pineda, J. R., Grange, G., Bryson, E. A., Guillermier, M., Hirsch, E., Hantraye, P. et al. (2006). Cystamine and cysteamine increase brain levels of BDNF in Huntington disease via HSJ1b and transglutaminase. *J. Clin. Invest.* 116, 1410-1424. 10.1172/JCI27607 16604191 PMC1430359

[DMM049447C15] Branco, J., Al-Ramahi, I., Ukani, L., Pérez, A. M., Fernandez-Funez, P., Rincón-Limas, D. and Botas, J. (2008). Comparative analysis of genetic modifiers in *Drosophila* points to common and distinct mechanisms of pathogenesis among polyglutamine diseases. *Hum. Mol. Genet.* 17, 376-390. 10.1093/hmg/ddm315 17984172

[DMM049447C16] Brehme, M. and Voisine, C. (2016). Model systems of protein-misfolding diseases reveal chaperone modifiers of proteotoxicity. *Dis. Model. Mech.* 9, 823-838. 10.1242/dmm.024703 27491084 PMC5007983

[DMM049447C17] Brehme, M., Voisine, C., Rolland, T., Wachi, S., Soper, J. H., Zhu, Y., Orton, K., Villella, A., Garza, D., Vidal, M. et al. (2014). A chaperome subnetwork safeguards proteostasis in aging and neurodegenerative disease. *Cell Rep.* 9, 1135-1150. 10.1016/j.celrep.2014.09.042 25437566 PMC4255334

[DMM049447C18] Bulthuis, N., Spontak, K. R., Kleeman, B. and Cavanaugh, D. J. (2019). Neuronal activity in non-LNv clock cells is required to produce free-running rest:activity rhythms in *Drosophila*. *J. Biol. Rhythms* 34, 249-271. 10.1177/0748730419841468 30994046 PMC7153773

[DMM049447C19] Carter, B., Justin, H. S., Gulick, D. and Gamsby, J. J. (2021). The molecular clock and neurodegenerative disease: a stressful time. *Front. Mol. Biosci.* 8, 644747. 10.3389/fmolb.2021.644747 33889597 PMC8056266

[DMM049447C20] Chai, Y., Koppenhafer, S. L., Bonini, N. M. and Paulson, H. L. (1999). Analysis of the role of Heat Shock Protein (Hsp) molecular chaperones in polyglutamine disease. *J. Neurosci.* 19, 10338-10347. 10.1523/JNEUROSCI.19-23-10338.1999 10575031 PMC6782415

[DMM049447C21] Chan, H. Y. E., Warrick, J. M., Gray-Board, G. L., Paulson, H. L. and Bonini, N. M. (2000). Mechanisms of chaperone suppression of polyglutamine disease: selectivity, synergy and modulation of protein solubility in *Drosophila*. *Hum. Mol. Genet.* 9, 2811-2820. 10.1093/hmg/9.19.2811 11092757

[DMM049447C22] Chuang, J.-Z., Zhou, H., Zhu, M., Li, S.-H., Li, X.-J. and Sung, C.-H. (2002). Characterization of a brain-enriched chaperone, MRJ, that inhibits Huntingtin aggregation and toxicity independently. *J. Biol. Chem.* 277, 19831-19838. 10.1074/jbc.M109613200 11896048

[DMM049447C23] Colwell, C. S. (2021). Defining circadian disruption in neurodegenerative disorders. *J. Clin. Invest.* 131, e148288. 10.1172/JCI148288 34596047 PMC8483739

[DMM049447C24] Deane, C. A. S. and Brown, I. R. (2016). Induction of heat shock proteins in differentiated human neuronal cells following co-application of celastrol and arimoclomol. *Cell Stress Chaperones* 21, 837-848. 10.1007/s12192-016-0708-2 27273088 PMC5003800

[DMM049447C25] Delventhal, R., O'Connor, R. M., Pantalia, M. M., Ulgherait, M., Kim, H. X., Basturk, M. K., Canman, J. C. and Shirasu-Hiza, M. (2019). Dissection of central clock function in *Drosophila* through cell-specific CRISPR-mediated clock gene disruption. *eLife* 8, e48308. 10.7554/eLife.48308 PMC6794090 31613218

[DMM049447C26] Desvergne, A. and Friguet, B. (2017). Circadian rhythms and proteostasis in aging. *Healthy Ageing and Longevity Circadian Rhythms and Their Impact on Aging* 7, 163-191. 10.1007/978-3-319-64543-8_8

[DMM049447C27] Dukay, B., Csoboz, B. and Tóth, M. E. (2019). Heat-Shock proteins in neuroinflammation. *Front. Pharmacol.* 10, 920. 10.3389/fphar.2019.00920 31507418 PMC6718606

[DMM049447C28] Edgar, R. S., Green, E. W., Zhao, Y., van Ooijen, G., Olmedo, M., Qin, X., Xu, Y., Pan, M., Valekunja, U. K., Feeney, K. A. et al. (2012). Peroxiredoxins are conserved markers of circadian rhythms. *Nature* 485, 459-464. 10.1038/nature11088 22622569 PMC3398137

[DMM049447C29] Escusa-Toret, S., Vonk, W. I. M. and Frydman, J. (2013). Spatial sequestration of misfolded proteins by a dynamic chaperone pathway enhances cellular fitness during stress. *Nat. Cell Biol.* 15, 1231-1243. 10.1038/ncb2838 24036477 PMC4121856

[DMM049447C30] Fifel, K. and Videnovic, A. (2020). Circadian alterations in patients with neurodegenerative diseases: Neuropathological basis of underlying network mechanisms. *Neurobiol. Dis.* 144, 105029. 10.1016/j.nbd.2020.105029 32736083

[DMM049447C31] Gamerdinger, M., Kaya, A. M., Wolfrum, U., Clement, A. M. and Behl, C. (2011). BAG3 mediates chaperone-based aggresome-targeting and selective autophagy of misfolded proteins. *EMBO Rep.* 12, 149-156. 10.1038/embor.2010.203 21252941 PMC3049430

[DMM049447C32] Gao, X., Carroni, M., Nussbaum-Krammer, C., Mogk, A., Nillegoda, N. B., Szlachcic, A., Guilbride, D. L., Saibil, H. R., Mayer, Matthias, P. et al. (2015). Human Hsp70 disaggregase reverses Parkinson's-linked α-Synuclein amyloid fibrils. *Mol. Cell* 59, 781-793. 10.1016/j.molcel.2015.07.012 26300264 PMC5072489

[DMM049447C33] García-Mata, R., Bebök, Z., Sorscher, E. J. and Sztul, E. S. (1999). Characterization and dynamics of aggresome formation by a cytosolic GFP-chimera. *J. Cell Biol.* 146, 1239-1254. 10.1083/jcb.146.6.1239 10491388 PMC2156127

[DMM049447C34] Gibbs, S. J., Barren, B., Beck, K. E., Proft, J., Zhao, X., Noskova, T., Braun, A. P., Artemyev, N. O. and Braun, J. E. A. (2009). Hsp40 couples with the CSPalpha chaperone complex upon induction of the heat shock response. *PLoS ONE* 4, e4595. 10.1371/journal.pone.0004595 19242542 PMC2643527

[DMM049447C35] Gitler, A. D., Dhillon, P. and Shorter, J. (2017). Neurodegenerative disease: models, mechanisms, and a new hope. *Dis. Model. Mech.* 10, 499-502. 10.1242/dmm.030205 28468935 PMC5451177

[DMM049447C36] Goodman, A. O. G., Rogers, L., Pilsworth, S., McAllister, C. J., Shneerson, J. M., Morton, A. J. and Barker, R. A. (2011). Asymptomatic sleep abnormalities are a common early feature in patients with Huntington's disease. *Curr. Neurol. Neurosci. Rep.* 11, 211-217. 10.1007/s11910-010-0163-x 21103960

[DMM049447C37] Gorenberg, E. L. and Chandra, S. S. (2017). The role of co-chaperones in synaptic proteostasis and neurodegenerative disease. *Front. Neurosci.* 11, 248. 10.3389/fnins.2017.00248 28579939 PMC5437171

[DMM049447C38] Grima, B., Chélot, E., Xia, R. and Rouyer, F. (2004). Morning and evening peaks of activity rely on different clock neurons of the *Drosophila* brain. *Nature* 431, 869-873. 10.1038/nature02935 15483616

[DMM049447C39] Gunawardena, S., Her, L.-S., Brusch, R. G., Laymon, R. A., Niesman, I. R., Gordesky-Gold, B., Sintasath, L., Bonini, N. M. and Goldstein, L. S. B. (2003). Disruption of axonal transport by loss of huntingtin or expression of pathogenic polyQ proteins in *Drosophila*. *Neuron* 40, 25-40. 10.1016/S0896-6273(03)00594-4 14527431

[DMM049447C40] Gusella, J. F. and MacDonald, M. E. (2006). Huntington's disease: seeing the pathogenic process through a genetic lens. *Trends Biochem. Sci.* 31, 533-540. 10.1016/j.tibs.2006.06.009 16829072

[DMM049447C41] Guzhova, I. V., Lazarev, V. F., Kaznacheeva, A. V., Ippolitova, M. V., Muronetz, V. I., Kinev, A. V. and Margulis, B. A. (2011). Novel mechanism of Hsp70 chaperone-mediated prevention of polyglutamine aggregates in a cellular model of Huntington disease. *Hum. Mol. Genet.* 20, 3953-3963. 10.1093/hmg/ddr314 21775503

[DMM049447C42] Hageman, J., Rujano, M. A., van Waarde, M. A. W. H., Kakkar, V., Dirks, R. P., Govorukhina, N., Oosterveld-Hut, H. M. J., Lubsen, N. H. and Kampinga, H. H. (2010). A DNAJB chaperone subfamily with HDAC-dependent activities suppresses toxic protein aggregation. *Mol. Cell* 37, 355-369. 10.1016/j.molcel.2010.01.001 20159555

[DMM049447C43] Hansson, O., Nylandsted, J., Castilho, R. F., Leist, M., Jäättelä, M. and Brundin, P. (2003). Overexpression of heat shock protein 70 in R6/2 Huntington's disease mice has only modest effects on disease progression. *Brain Res.* 970, 47-57. 10.1016/S0006-8993(02)04275-0 12706247

[DMM049447C44] Hartl, F. U. and Hayer-Hartl, M. (2009). Converging concepts of protein folding in vitro and in vivo. *Nat. Struct. Mol. Biol.* 16, 574-581. 10.1038/nsmb.1591 19491934

[DMM049447C45] Hay, D. G. (2004). Progressive decrease in chaperone protein levels in a mouse model of Huntington's disease and induction of stress proteins as a therapeutic approach. *Hum. Mol. Genet.* 13, 1389-1405. 10.1093/hmg/ddh144 15115766

[DMM049447C46] Helfrich-Förster, C. (1995). The period clock gene is expressed in central nervous system neurons which also produce a neuropeptide that reveals the projections of circadian pacemaker cells within the brain of *Drosophila melanogaster*. *Proc. Natl. Acad. Sci. USA* 92, 612-616. 10.1073/pnas.92.2.612 7831339 PMC42792

[DMM049447C47] Helfrich-Forster, C. (1997). Development of pigment-dispersing hormone-immunoreactive neurons in the nervous system of *Drosophila melanogaster*. *J. Comp. Neurol.* 380, 335-354. 10.1002/(SICI)1096-9861(19970414)380:3<335::AID-CNE4>3.0.CO;2-3 9087517

[DMM049447C48] Hipp, M. S., Kasturi, P. and Hartl, F. U. (2019). The proteostasis network and its decline in ageing. *Nat. Rev. Mol. Cell Biol.* 20, 421-435. 10.1038/s41580-019-0101-y 30733602

[DMM049447C49] Hood, S. and Amir, S. (2017). Neurodegeneration and the circadian clock. *Front. Aging Neurosci.* 9, 170. 10.3389/fnagi.2017.00170 28611660 PMC5447688

[DMM049447C50] Houl, J. H., Ng, F., Taylor, P. and Hardin, P. E. (2008). CLOCK expression identifies developing circadian oscillator neurons in the brains of *Drosophila* embryos. *BMC Neurosci.* 9, 119. 10.1186/1471-2202-9-119 19094242 PMC2628352

[DMM049447C163] Hung, H. C., Kay, S. A. and Weber, F. (2009) HSP90, a capacitor of behavioral variation. *J. Biol. Rhythms* 24, 183-192. 10.1177/0748730409333171 19465695

[DMM049447C51] Jäättelä, M. (1995). Over-expression of hsp70 confers tumorigenicity to mouse fibrosarcoma cells. *Int. J. Cancer* 60, 689-693. 10.1002/ijc.2910600520 7860144

[DMM049447C52] Jana, N. R. (2000). Polyglutamine length-dependent interaction of Hsp40 and Hsp70 family chaperones with truncated N-terminal huntingtin: their role in suppression of aggregation and cellular toxicity. *Hum. Mol. Genet.* 9, 2009-2018. 10.1093/hmg/9.13.2009 10942430

[DMM049447C53] Jiang, Y., Lv, H., Liao, M., Xu, X., Huang, S., Tan, H., Peng, T., Zhang, Y. and Li, H. (2012). GRP78 counteracts cell death and protein aggregation caused by mutant huntingtin proteins. *Neurosci. Lett.* 516, 182-187. 10.1016/j.neulet.2012.03.074 22490889

[DMM049447C54] Johnston, H. E. and Samant, R. S. (2021). Alternative systems for misfolded protein clearance: life beyond the proteasome. *FEBS J.* 288, 4464-4487. 10.1111/febs.15617 33135311

[DMM049447C55] Johnston, J. A., Ward, C. L. and Kopito, R. R. (1998). Aggresomes: a cellular response to misfolded proteins. *J. Cell Biol.* 143, 1883-1898. 10.1083/jcb.143.7.1883 9864362 PMC2175217

[DMM049447C56] Junn, E., Lee, S. S., Suhr, U. T. and Mouradian, M. M. (2002). Parkin accumulation in aggresomes due to proteasome impairment. *J. Biol. Chem.* 277, 47870-47877. 10.1074/jbc.M203159200 12364339

[DMM049447C57] Juste, Y. R., Kaushik, S., Bourdenx, M., Aflakpui, R., Bandyopadhyay, S., Garcia, F., Diaz, A., Lindenau, K., Tu, V., Krause, G. J. et al. (2021). Reciprocal regulation of chaperone-mediated autophagy and the circadian clock. *Nat. Cell Biol.* 23, 1255-1270. 10.1038/s41556-021-00800-z 34876687 PMC8688252

[DMM049447C58] Kakkar, V., Månsson, C., de Mattos, E. P., Bergink, S., van der Zwaag, M., van Waarde, M., Kloosterhuis, N. J., Melki, R., van Cruchten, R. T. P., Al-Karadaghi, S. et al. (2016). The S/T-rich motif in the DNAJB6 chaperone delays polyglutamine aggregation and the onset of disease in a mouse model. *Mol. Cell* 62, 272-283. 10.1016/j.molcel.2016.03.017 27151442

[DMM049447C59] Kalmar, B. and Greensmith, L. (2009). Induction of heat shock proteins for protection against oxidative stress. *Adv. Drug Deliv. Rev.* 61, 310-318. 10.1016/j.addr.2009.02.003 19248813

[DMM049447C60] Kampinga, H. H. and Craig, E. A. (2010). The HSP70 chaperone machinery: J proteins as drivers of functional specificity. *Nat. Rev. Mol. Cell Biol.* 11, 579-592. 10.1038/nrm2941 20651708 PMC3003299

[DMM049447C61] Kampinga, H. H. and Bergink, S. (2016). Heat shock proteins as potential targets for protective strategies in neurodegeneration. *Lancet Neurol.* 15, 748-759. 10.1016/S1474-4422(16)00099-5 27106072

[DMM049447C62] Kampinga, H. H., Andreasson, C., Barducci, A., Cheetham, M. E., Cyr, D., Emanuelsson, C., Genevaux, P., Gestwicki, J. E., Goloubinoff, P., Huerta-Cepas, J. et al. (2019). Function, evolution, and structure of J-domain proteins. *Cell Stress Chaperones* 24, 7-15. 10.1007/s12192-018-0948-4 30478692 PMC6363617

[DMM049447C63] Karpuj, M. V., Becher, M. W., Springer, J. E., Chabas, D., Youssef, S., Pedotti, R., Mitchell, D. and Steinman, L. (2002). Prolonged survival and decreased abnormal movements in transgenic model of Huntington disease, with administration of the transglutaminase inhibitor cystamine. *Nat. Med.* 8, 143-149. 10.1038/nm0202-143 11821898

[DMM049447C64] Kennedy, D., Jäger, R., Mosser, D. D. and Samali, A. (2014). Regulation of apoptosis by heat shock proteins. *IUBMB Life* 66, 327-338. 10.1002/iub.1274 24861574

[DMM049447C65] Kim, S., Nollen, E. A. A., Kitagawa, K., Bindokas, V. P. and Morimoto, R. I. (2002). Polyglutamine protein aggregates are dynamic. *Nat. Cell Biol.* 4, 826-831. 10.1038/ncb863 12360295

[DMM049447C66] Kim, Y. E., Hipp, M. S., Bracher, A., Hayer-Hartl, M. and Ulrich Hartl, F. (2013). Molecular chaperone functions in protein folding and proteostasis. *Annu. Rev. Biochem.* 82, 323-355. 10.1146/annurev-biochem-060208-092442 23746257

[DMM049447C67] Kim, M., Subramanian, M., Cho, Y.-H., Kim, G.-H., Lee, E. and Park, J.-J. (2018). Short-term exposure to dim light at night disrupts rhythmic behaviors and causes neurodegeneration in fly models of tauopathy and Alzheimer's disease. *Biochem. Biophys. Res. Commun.* 495, 1722-1729. 10.1016/j.bbrc.2017.12.021 29217196

[DMM049447C68] Kobayashi, Y., Kume, A., Li, M., Doyu, M., Hata, M., Ohtsuka, K. and Sobue, G. (2000). Chaperones Hsp70 and Hsp40 suppress aggregate formation and apoptosis in cultured neuronal cells expressing truncated androgen receptor protein with expanded polyglutamine tract. *J. Biol. Chem.* 275, 8772-8778. 10.1074/jbc.275.12.8772 10722721

[DMM049447C69] Kopito, R. R. (2000). Aggresomes, inclusion bodies and protein aggregation. *Trends Cell Biol.* 10, 524-530. 10.1016/S0962-8924(00)01852-3 11121744

[DMM049447C70] Kostadinov, B., Lee Pettibone, H., Bell, E. V., Zhou, X., Pranevicius, A., Shafer, O. T. and Fernandez, M. P. (2021). Open-source computational framework for studying *Drosophila* behavioral phase. *STAR Protoc.* 2, 100285. 10.1016/j.xpro.2020.100285 33532734 PMC7829270

[DMM049447C71] Krishnan, N., Rakshit, K., Chow, E. S., Wentzell, J. S., Kretzschmar, D. and Giebultowicz, J. M. (2012). Loss of circadian clock accelerates aging in neurodegeneration-prone mutants. *Neurobiol. Dis.* 45, 1129-1135. 10.1016/j.nbd.2011.12.034 22227001 PMC3291167

[DMM049447C72] Kudo, T., Schroeder, A., Loh, D. H., Kuljis, D., Jordan, M. C., Roos, K. P. and Colwell, C. S. (2011). Dysfunctions in circadian behavior and physiology in mouse models of Huntington's disease. *Exp. Neurol.* 228, 80-90. 10.1016/j.expneurol.2010.12.011 21184755 PMC4346330

[DMM049447C73] Kula-Eversole, E., Nagoshi, E., Shang, Y., Rodriguez, J., Allada, R. and Rosbash, M. (2010). Surprising gene expression patterns within and between PDF-containing circadian neurons in *Drosophila*. *Proc. Natl. Acad. Sci. USA* 107, 13497-13502. 10.1073/pnas.1002081107 20624977 PMC2922133

[DMM049447C74] Kuo, Y., Ren, S., Lao, U., Edgar, B. A. and Wang, T. (2013). Suppression of polyglutamine protein toxicity by co-expression of a heat-shock protein 40 and a heat-shock protein 110. *Cell Death Dis.* 4, e833. 10.1038/cddis.2013.351 24091676 PMC3824661

[DMM049447C75] Labbadia, J., Novoselov, S. S., Bett, J. S., Weiss, A., Paganetti, P., Bates, G. P. and Cheetham, M. E. (2012). Suppression of protein aggregation by chaperone modification of high molecular weight complexes. *Brain* 135, 1180-1196. 10.1093/brain/aws022 22396390 PMC3326252

[DMM049447C76] Lassonde, M., Candel, S., Hacker, J., Quadrio-Curzio, A., Onishi, T., Ramakrishnan, V. and McNutt, M. (2017). The challenge of neurodegenerative diseases in an aging population. *Trends Sci.* 22, 6_92-6_93. 10.5363/tits.22.6_92

[DMM049447C77] Lauretti, E., Di Meco, A., Merali, S. and Praticò, D. (2017). Circadian rhythm dysfunction: a novel environmental risk factor for Parkinson's disease. *Mol. Psychiatry* 22, 280-286. 10.1038/mp.2016.47 27046648

[DMM049447C78] Lazar, A. S., Panin, F., Goodman, A. O. G., Lazic, S. E., Lazar, Z. I., Mason, S. L., Rogers, L., Murgatroyd, P. R., Watson, L. P. E., Singh, P. et al. (2015). Sleep deficits but no metabolic deficits in premanifest Huntington's disease. *Ann. Neurol.* 78, 630-648. 10.1002/ana.24495 26224419 PMC4832311

[DMM049447C79] Lebreton, F., Cayzac, S., Pietropaolo, S., Jeantet, Y. and Cho, Y. H. (2015). Sleep physiology alterations precede plethoric phenotypic changes in R6/1 Huntington's disease mice. *PLoS ONE* 10, e0126972. 10.1371/journal.pone.0126972 25966356 PMC4428700

[DMM049447C80] Lee, W.-C. M., Yoshihara, M. and Littleton, J. T. (2004). Cytoplasmic aggregates trap polyglutamine-containing proteins and block axonal transport in a *Drosophila* model of Huntington's disease. *Proc. Natl. Acad. Sci. USA* 101, 3224-3229. 10.1073/pnas.0400243101 14978262 PMC365771

[DMM049447C81] Leng, Y., Musiek, E. S., Hu, K., Cappuccio, F. P. and Yaffe, K. (2019). Association between circadian rhythms and neurodegenerative diseases. *Lancet Neurol.* 18, 307-318. 10.1016/S1474-4422(18)30461-7 30784558 PMC6426656

[DMM049447C82] Li, S., Shui, K., Zhang, Y., Lv, Y., Deng, W., Ullah, S., Zhang, L. and Xue, Y. (2017). CGDB: a database of circadian genes in eukaryotes. *Nucleic Acids Res.* 45, D397-D403. 27789706 10.1093/nar/gkw1028 PMC5210527

[DMM049447C83] Lotz, G. P., Legleiter, J., Aron, R., Mitchell, E. J., Huang, S.-Y., Ng, C., Glabe, C., Thompson, L. M. and Muchowski, P. J. (2010). Hsp70 and Hsp40 functionally interact with soluble mutant huntingtin oligomers in a classic ATP-dependent reaction cycle. *J. Biol. Chem.* 285, 38183-38193. 10.1074/jbc.M110.160218 20864533 PMC2992252

[DMM049447C84] Ma, D., Przybylski, D., Abruzzi, K. C., Schlichting, M., Li, Q., Long, X. and Rosbash, M. (2021). A transcriptomic taxonomy of *Drosophila* circadian neurons around the clock. *eLife* 10, e63056. 10.7554/eLife.63056 PMC7837698 33438579

[DMM049447C85] Maheshwari, M., Bhutani, S., Das, A., Mukherjee, R., Sharma, A., Kino, Y., Nukina, N. and Jana, N. R. (2014). Dexamethasone induces heat shock response and slows down disease progression in mouse and fly models of Huntington's disease. *Hum. Mol. Genet.* 23, 2737-2751. 10.1093/hmg/ddt667 24381308

[DMM049447C86] Mannini, B. and Chiti, F. (2017). Chaperones as suppressors of protein misfolded oligomer toxicity. *Front. Mol. Neurosci.* 10, 98. 10.3389/fnmol.2017.00098 28424588 PMC5380756

[DMM049447C87] Månsson, C., Kakkar, V., Monsellier, E., Sourigues, Y., Härmark, J., Kampinga, H. H., Melki, R. and Emanuelsson, C. (2013). DNAJB6 is a peptide-binding chaperone which can suppress amyloid fibrillation of polyglutamine peptides at substoichiometric molar ratios. *Cell Stress Chaperones* 19, 227-239. 10.1007/s12192-013-0448-5 23904097 PMC3933622

[DMM049447C88] Maywood, E. S., Fraenkel, E., McAllister, C. J., Wood, N., Reddy, A. B., Hastings, M. H. and Morton, A. J. (2010). Disruption of peripheral circadian timekeeping in a mouse model of Huntington's disease and its restoration by temporally scheduled feeding. *J. Neurosci.* 30, 10199-10204. 10.1523/JNEUROSCI.1694-10.2010 20668203 PMC6633357

[DMM049447C89] McDonald, J. H. (2014). *Handbook of Biological Statistics*. Baltimore: Sparky House Publishing.

[DMM049447C90] McLear, J. A., Lebrecht, D., Messer, A. and Wolfgang, W. J. (2008). Combinational approach of intrabody with enhanced Hsp70 expression addresses multiple pathologies in a fly model of Huntington's disease. *FASEB J.* 22, 2003-2011. 10.1096/fj.07-099689 18199697

[DMM049447C91] Means, J. C., Venkatesan, A., Gerdes, B., Fan, J.-Y., Bjes, E. S. and Price, J. L. (2015). *Drosophila* spaghetti and doubletime link the circadian clock and light to caspases, apoptosis and tauopathy. *PLoS Genet.* 11, e1005171. 10.1371/journal.pgen.1005171 25951229 PMC4423883

[DMM049447C92] Mehra, A., Baker, C. L., Loros, J. J. and Dunlap, J. C. (2009). Post-translational modifications in circadian rhythms. *Trends Biochem. Sci.* 34, 483-490. 10.1016/j.tibs.2009.06.006 19740663 PMC2765057

[DMM049447C93] Menzies, F. M., Fleming, A., Caricasole, A., Bento, C. F., Andrews, S. P., Ashkenazi, A., Füllgrabe, J., Jackson, A., Jimenez Sanchez, M., Karabiyik, C. et al. (2017). Autophagy and neurodegeneration: Pathogenic mechanisms and therapeutic opportunities. *Neuron* 93, 1015-1034. 10.1016/j.neuron.2017.01.022 28279350

[DMM049447C94] Metaxakis, A., Ploumi, C. and Tavernarakis, N. (2018). Autophagy in age-associated neurodegeneration. *Cells* 7, 37. 10.3390/cells7050037 PMC5981261 29734735

[DMM049447C95] Miller, S. B. M., Ho, C. T., Winkler, J., Khokhrina, M., Neuner, A., Mohamed, M. Y. H., Guilbride, D. L., Richter, K., Lisby, M., Schiebel, E. et al. (2015). Compartment-specific aggregases direct distinct nuclear and cytoplasmic aggregate deposition. *EMBO J.* 34, 778-797. 10.15252/embj.201489524 25672362 PMC4369314

[DMM049447C96] Morton, A. J., Wood, N. I., Hastings, M. H., Hurelbrink, C., Barker, R. A. and Maywood, E. S. (2005). Disintegration of the sleep-wake cycle and circadian timing in Huntington's disease. *J. Neurosci.* 25, 157-163. 10.1523/JNEUROSCI.3842-04.2005 15634777 PMC6725210

[DMM049447C97] Morton, A. J., Rudiger, S. R., Wood, N. I., Sawiak, S. J., Brown, G. C., McLaughlan, C. J., Kuchel, T. R., Snell, R. G., Faull, R. L. M. and Bawden, C. S. (2014). Early and progressive circadian abnormalities in Huntington's disease sheep are unmasked by social environment. *Hum. Mol. Genet.* 23, 3375-3383. 10.1093/hmg/ddu047 24488771

[DMM049447C98] Muchowski, P. J., Schaffar, G., Sittler, A., Wanker, E. E., Hayer-Hartl, M. K. and Hartl, F. U. (2000). Hsp70 and Hsp40 chaperones can inhibit self-assembly of polyglutamine proteins into amyloid-like fibrils. *Proc. Natl. Acad. Sci. USA* 97, 7841-7846. 10.1073/pnas.140202897 10859365 PMC16632

[DMM049447C99] Mugat, B., Parmentier, M.-L., Bonneaud, N., Chan, H. Y. E. and Maschat, F. (2008). Protective role of Engrailed in a *Drosophila* model of Huntington's disease. *Hum. Mol. Genet.* 17, 3601-3616. 10.1093/hmg/ddn255 18718937

[DMM049447C100] Na, D., Rouf, M., O'Kane, C. J., Rubinsztein, D. C. and Gsponer, J. (2013). NeuroGeM, a knowledgebase of genetic modifiers in neurodegenerative diseases. *BMC Med. Genomics* 6, 52. 10.1186/1755-8794-6-52 24229347 PMC3833180

[DMM049447C101] Nillegoda, N. B., Wentink, A. S. and Bukau, B. (2018). Protein disaggregation in multicellular organisms. *Trends Biochem. Sci.* 43, 285-300. 10.1016/j.tibs.2018.02.003 29501325

[DMM049447C102] Nitabach, M. N., Wu, Y., Sheeba, V., Lemon, W. C., Strumbos, J., Zelensky, P. K., White, B. H. and Holmes, T. C. (2006). Electrical hyperexcitation of lateral ventral pacemaker neurons desynchronizes downstream circadian oscillators in the fly circadian circuit and induces multiple behavioral periods. *J. Neurosci.* 26, 479-489. 10.1523/JNEUROSCI.3915-05.2006 16407545 PMC2597197

[DMM049447C103] Nollen, E. A. A., Salomons, F. A., Brunsting, J. F., van der Want, J. J. L., Sibon, O. C. M. and Kampinga, H. H. (2001). Dynamic changes in the localization of thermally unfolded nuclear proteins associated with chaperone-dependent protection. *Proc. Natl. Acad. Sci. USA* 98, 12038-12043. 10.1073/pnas.201112398 11572931 PMC59763

[DMM049447C104] Nylandsted, J., Wick, W., Hirt, U. A., Brand, K., Rohde, M., Leist, M., Weller, M. and Jaattela, M. (2002). Eradication of glioblastoma, and breast and colon carcinoma xenografts by Hsp70 depletion. *Cancer Res.* 62, 7139-7142. 12499245

[DMM049447C105] Ormsby, A. R., Ramdzan, Y. M., Mok, Y.-F., Jovanoski, K. D. and Hatters, D. M. (2013). A platform to view huntingtin exon 1 aggregation flux in the cell reveals divergent influences from chaperones hsp40 and hsp70. *J. Biol. Chem.* 288, 37192-37203. 10.1074/jbc.M113.486944 24196953 PMC3873573

[DMM049447C106] Ouk, K., Aungier, J. and Morton, A. J. (2017). Prolonged day length exposure improves circadian deficits and survival in a transgenic mouse model of Huntington's disease. *Neurobiol. Sleep Circadian Rhythms* 2, 27-38. 10.1016/j.nbscr.2016.11.004 31236493 PMC6575567

[DMM049447C107] Pallier, P. N., Maywood, E. S., Zheng, Z., Chesham, J. E., Inyushkin, A. N., Dyball, R., Hastings, M. H. and Morton, A. J. (2007). Pharmacological imposition of sleep slows cognitive decline and reverses dysregulation of circadian gene expression in a transgenic mouse model of Huntington's disease. *J. Neurosci.* 27, 7869-7878. 10.1523/JNEUROSCI.0649-07.2007 17634381 PMC6672877

[DMM049447C108] Park, J. H., Helfrich-Forster, C., Lee, G., Liu, L., Rosbash, M. and Hall, J. C. (2000). Differential regulation of circadian pacemaker output by separate clock genes in *Drosophila*. *Proc. Natl. Acad. Sci. USA* 97, 3608-3613. 10.1073/pnas.97.7.3608 10725392 PMC16287

[DMM049447C109] Pfeiffenberger, C., Lear, B. C., Keegan, K. P. and Allada, R. (2010). Processing circadian data collected from the *Drosophila* Activity Monitoring (DAM) System. *Cold Spring Harb. Protoc.* 2010, pdb prot5519. 10.1101/pdb.prot5519 21041392

[DMM049447C110] Pickard, A., Chang, J., Alachkar, N., Calverley, B., Garva, R., Arvan, P., Meng, Q.-J. and Kadler, K. E. (2019). Preservation of circadian rhythms by the protein folding chaperone, BiP. *FASEB J.* 33, 7479-7489. 10.1096/fj.201802366RR 30888851 PMC6529331

[DMM049447C111] Popiel, H. A., Takeuchi, T., Fujita, H., Yamamoto, K., Ito, C., Yamane, H., Muramatsu, S.-I., Toda, T., Wada, K. and Nagai, Y. (2012). Hsp40 gene therapy exerts therapeutic effects on polyglutamine disease mice via a non-cell autonomous mechanism. *PLoS ONE* 7, e51069. 10.1371/journal.pone.0051069 23226463 PMC3511362

[DMM049447C112] Prakash, P., Nambiar, A. and Sheeba, V. (2017). Oscillating PDF in termini of circadian pacemaker neurons and synchronous molecular clocks in downstream neurons are not sufficient for sustenance of activity rhythms in constant darkness. *PLoS ONE* 12, e0175073. 10.1371/journal.pone.0175073 28558035 PMC5448722

[DMM049447C113] Renn, S. C. P., Park, J. H., Rosbash, M., Hall, J. C. and Taghert, P. H. (1999). A *pdf* neuropeptide gene mutation and ablation of PDF neurons each cause severe abnormalities of behavioral circadian rhythms in *Drosophila*. *Cell* 99, 791-802. 10.1016/S0092-8674(00)81676-1 10619432

[DMM049447C114] Rey, G., Milev, N. B., Valekunja, U. K., Ch, R., Ray, S., Silva Dos Santos, M., Nagy, A. D., Antrobus, R., MacRae, J. I. and Reddy, A. B. (2018). Metabolic oscillations on the circadian time scale in *Drosophila* cells lacking clock genes. *Mol. Syst. Biol.* 14, e8376. 10.15252/msb.20188376 30072421 PMC6078164

[DMM049447C115] Rezaval, C., Werbajh, S. and Ceriani, M. F. (2007). Neuronal death in *Drosophila* triggered by GAL4 accumulation. *Eur. J. Neurosci.* 25, 683-694. 10.1111/j.1460-9568.2007.05317.x 17313569

[DMM049447C116] Rujano, M. A., Kampinga, H. H. and Salomons, F. A. (2007). Modulation of polyglutamine inclusion formation by the Hsp70 chaperone machine. *Exp. Cell Res.* 313, 3568-3578. 10.1016/j.yexcr.2007.07.034 17822698

[DMM049447C117] Ryzhikov, M., Ehlers, A., Steinberg, D., Xie, W., Oberlander, E., Brown, S., Gilmore, P. E., Townsend, R. R., Lane, W. S., Dolinay, T. et al. (2019). Diurnal rhythms spatially and temporally organize autophagy. *Cell Rep.* 26, 1880-1892.e6. 10.1016/j.celrep.2019.01.072 30759397 PMC6442472

[DMM049447C118] Sang, T.-K., Li, C., Liu, W., Rodriguez, A., Abrams, J. M., Zipursky, S. L. and Jackson, G. R. (2005). Inactivation of *Drosophila* Apaf-1 related killer suppresses formation of polyglutamine aggregates and blocks polyglutamine pathogenesis. *Hum. Mol. Genet.* 14, 357-372. 10.1093/hmg/ddi032 15590702

[DMM049447C119] Schlichting, M., Diaz, M. M., Xin, J. and Rosbash, M. (2019). Neuron-specific knockouts indicate the importance of network communication to *Drosophila* rhythmicity. *eLife* 8, e48301. 10.7554/eLife.48301 PMC6794074 31613223

[DMM049447C120] Schneider, C. A., Rasband, W. S. and Eliceiri, K. W. (2012). NIH Image to ImageJ: 25 years of image analysis. *Nat. Methods* 9, 671-675. 10.1038/nmeth.2089 22930834 PMC5554542

[DMM049447C121] Schneider, R., Linka, R. M. and Reinke, H. (2014). HSP90 affects the stability of BMAL1 and circadian gene expression. *J. Biol. Rhythms* 29, 87-96. 10.1177/0748730414523559 24682203

[DMM049447C122] Scior, A., Buntru, A., Arnsburg, K., Ast, A., Iburg, M., Juenemann, K., Pigazzini, M. L., Mlody, B., Puchkov, D., Priller, J. et al. (2018). Complete suppression of Htt fibrilization and disaggregation of Htt fibrils by a trimeric chaperone complex. *EMBO J.* 37, 282-299. 10.15252/embj.201797212 29212816 PMC5770855

[DMM049447C123] Shafer, O. T. and Taghert, P. H. (2009). RNA-interference knockdown of *Drosophila* pigment dispersing factor in neuronal subsets: the anatomical basis of a neuropeptide's circadian functions. *PLoS One* 4, e8298. 10.1371/journal.pone.0008298 20011537 PMC2788783

[DMM049447C124] Shafer, O. T., Rosbash, M. and Truman, J. W. (2002). Sequential nuclear accumulation of the clock proteins Period and Timeless in the pacemaker neurons of *Drosophila melanogaster*. *J. Neurosci.* 22, 5946-5954. 10.1523/JNEUROSCI.22-14-05946.2002 12122057 PMC6757919

[DMM049447C125] Sharma, A. and Goyal, R. (2020). Long-term exposure to constant light induces dementia, oxidative stress and promotes aggregation of sub-pathological Abeta42 in Wistar rats. *Pharmacol. Biochem. Behav.* 192, 172892. 10.1016/j.pbb.2020.172892 32142744

[DMM049447C126] Sheeba, V., Fogle, K. J., Kaneko, M., Rashid, S., Chou, Y.-T., Sharma, V. K. and Holmes, T. C. (2008). Large ventral lateral neurons modulate arousal and sleep in *Drosophila*. *Curr. Biol.* 18, 1537-1545. 10.1016/j.cub.2008.08.033 18771923 PMC2597195

[DMM049447C127] Sheeba, V., Fogle, K. J. and Holmes, T. C. (2010). Persistence of morning anticipation behavior and high amplitude morning startle response following functional loss of small ventral lateral neurons in *Drosophila*. *PLoS ONE* 5, e11628. 10.1371/journal.pone.0011628 20661292 PMC2905440

[DMM049447C128] Shulman, J. M. and Feany, M. B. (2003). Genetic modifiers of tauopathy in *Drosophila*. *Genetics* 165, 1233-1242. 10.1093/genetics/165.3.1233 14668378 PMC1462852

[DMM049447C129] Sittler, A., Lurz, R., Lueder, G., Priller, J., Lehrach, H., Hayer-Hartl, M. K., Hartl, F. U. and Wanker, E. E. (2001). Geldanamycin activates a heat shock response and inhibits huntingtin aggregation in a cell culture model of Huntington's disease. *Hum. Mol. Genet.* 10, 1307-1315. 10.1093/hmg/10.12.1307 11406612

[DMM049447C130] Smarr, B., Cutler, T., Loh, D. H., Kudo, T., Kuljis, D., Kriegsfeld, L., Ghiani, C. A. and Colwell, C. S. (2019). Circadian dysfunction in the Q175 model of Huntington's disease: Network analysis. *J. Neurosci. Res.* 97, 1606-1623. 10.1002/jnr.24505 31359503 PMC7105276

[DMM049447C131] Soneson, C., Fontes, M., Zhou, Y., Denisov, V., Paulsen, J. S., Kirik, D., Petersen, A. and The Huntington Study Group PREDICT-HD investigators. (2010). Early changes in the hypothalamic region in prodromal Huntington disease revealed by MRI analysis. *Neurobiol. Dis.* 40, 531-543. 10.1016/j.nbd.2010.07.013 20682340 PMC2955781

[DMM049447C132] Sontag, E. M., Vonk, W. I. M. and Frydman, J. (2014). Sorting out the trash: the spatial nature of eukaryotic protein quality control. *Curr. Opin. Cell Biol.* 26, 139-146. 10.1016/j.ceb.2013.12.006 24463332 PMC4204729

[DMM049447C133] Specht, S., Miller, S. B. M., Mogk, A. and Bukau, B. (2011). Hsp42 is required for sequestration of protein aggregates into deposition sites in *Saccharomyces cerevisiae*. *J. Cell Biol.* 195, 617-629. 10.1083/jcb.201106037 22065637 PMC3257523

[DMM049447C134] Steffan, J. S., Bodai, L., Pallos, J., Poelman, M., McCampbell, A., Apostol, B. L., Kazantsev, A., Schmidt, E., Zhu, Y.-Z., Greenwald, M. et al. (2001). Histone deacetylase inhibitors arrest polyglutamine-dependent neurodegeneration in *Drosophila*. *Nature* 413, 739-743. 10.1038/35099568 11607033

[DMM049447C135] Stojkovic, K., Wing, S. S. and Cermakian, N. (2014). A central role for ubiquitination within a circadian clock protein modification code. *Front. Mol. Neurosci.* 7, 69. 10.3389/fnmol.2014.00069 25147498 PMC4124793

[DMM049447C136] Stoleru, D., Peng, Y., Agosto, J. and Rosbash, M. (2004). Coupled oscillators control morning and evening locomotor behaviour of *Drosophila*. *Nature* 431, 862-868. 10.1038/nature02926 15483615

[DMM049447C137] Sutton, L. M., Sanders, S. S., Butland, S. L., Singaraja, R. R., Franciosi, S., Southwell, A. L., Doty, C. N., Schmidt, M. E., Mui, K. K. N., Kovalik, V. et al. (2013). Hip14l-deficient mice develop neuropathological and behavioural features of Huntington disease. *Hum. Mol. Genet.* 22, 452-465. 10.1093/hmg/dds441 23077216

[DMM049447C138] Tagawa, K., Marubuchi, S., Qi, M.-L., Enokido, Y., Tamura, T., Inagaki, R., Murata, M., Kanazawa, I., Wanker, E. E. and Okazawa, H. (2007). The induction levels of heat shock protein 70 differentiate the vulnerabilities to mutant huntingtin among neuronal subtypes. *J. Neurosci.* 27, 868-880. 10.1523/JNEUROSCI.4522-06.2007 17251428 PMC6672912

[DMM049447C139] Tamaru, T., Hattori, M., Honda, K., Benjamin, I., Ozawa, T. and Takamatsu, K. (2011). Synchronization of circadian Per2 rhythms and HSF1-BMAL1:CLOCK interaction in mouse fibroblasts after short-term heat shock pulse. *PLoS ONE* 6, e24521. 10.1371/journal.pone.0024521 21915348 PMC3168500

[DMM049447C140] Tan, S. and Wong, E. (2017). Kinetics of protein aggregates disposal by aggrephagy. *Methods Enzymol.* 588, 245-281. 10.1016/bs.mie.2016.09.084 28237105

[DMM049447C141] Taylor, R. C. and Dillin, A. (2011). Aging as an event of proteostasis collapse. *Cold Spring Harbor Perspect. Biol.* 3, a004440. 10.1101/cshperspect.a004440 PMC3101847 21441594

[DMM049447C142] Tittelmeier, J., Nachman, E. and Nussbaum-Krammer, C. (2020a). Molecular chaperones: a double-edged sword in neurodegenerative diseases. *Front. Aging Neurosci.* 12, 581374. 10.3389/fnagi.2020.581374 33132902 PMC7572858

[DMM049447C143] Tittelmeier, J., Sandhof, C. A., Ries, H. M., Druffel-Augustin, S., Mogk, A., Bukau, B. and Nussbaum-Krammer, C. (2020b). The HSP110/HSP70 disaggregation system generates spreading-competent toxic alpha-synuclein species. *EMBO J.* 39, e103954. 10.15252/embj.2019103954 32449565 PMC7327497

[DMM049447C144] Toledo, M., Batista-Gonzalez, A., Merheb, E., Aoun, M. L., Tarabra, E., Feng, D., Sarparanta, J., Merlo, P., Botre, F., Schwartz, G. J. et al. (2018). Autophagy regulates the liver clock and glucose metabolism by degrading CRY1. *Cell Metab.* 28, 268-281.e4. 10.1016/j.cmet.2018.05.023 29937374 PMC6082686

[DMM049447C145] van Wamelen, D. J., Aziz, N. A., Anink, J. J., van Steenhoven, R., Angeloni, D., Fraschini, F., Jockers, R., Roos, R. A. and Swaab, D. F. (2013). Suprachiasmatic Nucleus neuropeptide expression in patients with Huntington's Disease. *Sleep* 36, 117-125. 10.5665/sleep.2314 23288978 PMC3524533

[DMM049447C146] Veleri, S., Brandes, C., Helfrich-Förster, C., Hall, J. C. and Stanewsky, R. (2003). A self-sustaining, light-entrainable circadian oscillator in the *Drosophila* brain. *Curr. Biol.* 13, 1758-1767. 10.1016/j.cub.2003.09.030 14561400

[DMM049447C147] Voysey, Z., Fazal, S. V., Lazar, A. S. and Barker, R. A. (2021). The sleep and circadian problems of Huntington's disease: when, why and their importance. *J. Neurol.* 268, 2275-2283. 10.1007/s00415-020-10334-3 33355880 PMC8179890

[DMM049447C148] Wacker, J. L., Huang, S.-Y., Steele, A. D., Aron, R., Lotz, G. P., Nguyen, Q., Giorgini, F., Roberson, E. D., Lindquist, S., Masliah, E. et al. (2009). Loss of Hsp70 exacerbates pathogenesis but not levels of fibrillar aggregates in a mouse model of Huntington's disease. *J. Neurosci.* 29, 9104-9114. 10.1523/JNEUROSCI.2250-09.2009 19605647 PMC2739279

[DMM049447C149] Wang, H.-B., Whittaker, D. S., Truong, D., Mulji, A. K., Ghiani, C. A., Loh, D. H. and Colwell, C. S. (2017). Blue light therapy improves circadian dysfunction as well as motor symptoms in two mouse models of Huntington's disease. *Neurobiol. Sleep Circadian Rhythms* 2, 39-52. 10.1016/j.nbscr.2016.12.002 31236494 PMC6575206

[DMM049447C150] Wang, X., Xu, Z., Cai, Y., Zeng, S., Peng, B., Ren, X., Yan, Y. and Gong, Z. (2020). Rheostatic balance of circadian rhythm and autophagy in metabolism and disease. *Front. Cell Dev. Biol.* 8, 616434. 10.3389/fcell.2020.616434 33330516 PMC7732583

[DMM049447C151] Westhoff, B., Chapple, J. P., van der Spuy, J., Höhfeld, J. and Cheetham, M. E. (2005). HSJ1 is a neuronal shuttling factor for the sorting of chaperone clients to the proteasome. *Curr. Biol.* 15, 1058-1064. 10.1016/j.cub.2005.04.058 15936278

[DMM049447C152] Whittaker, D. S., Loh, D. H., Wang, H.-B., Tahara, Y., Kuljis, D., Cutler, T., Ghiani, C. A., Shibata, S., Block, G. D. and Colwell, C. S. (2018). Circadian-based treatment strategy effective in the BACHD mouse model of Huntington's disease. *J. Biol. Rhythms* 33, 535-554. 10.1177/0748730418790401 30084274

[DMM049447C153] Wyttenbach, A. (2002). Heat shock protein 27 prevents cellular polyglutamine toxicity and suppresses the increase of reactive oxygen species caused by huntingtin. *Hum. Mol. Genet.* 11, 1137-1151. 10.1093/hmg/11.9.1137 11978772

[DMM049447C154] Wyttenbach, A. and Arrigo, A. P. (2009). The role of heat shock proteins during neurodegeneration in Alzheimer's, Parkinson's and Huntington's disease. In *Heat Shock Proteins in Neural Cells* (ed. C. Richter-Landsberg), pp. 81-99. Springer. 10.1007/978-0-387-39954-6_7

[DMM049447C155] Wyttenbach, A., Carmichael, J., Swartz, J., Furlong, R. A., Narain, Y., Rankin, J. and Rubinsztein, D. C. (2000). Effects of heat shock, heat shock protein 40 (HDJ-2), and proteasome inhibition on protein aggregation in cellular models of Huntington's disease. *Proc. Natl. Acad. Sci. USA* 97, 2898-2903. 10.1073/pnas.97.6.2898 10717003 PMC16027

[DMM049447C156] Xu, F., Kula-Eversole, E., Iwanaszko, M., Lim, C. and Allada, R. (2019a). Ataxin2 functions via CrebA to mediate Huntingtin toxicity in circadian clock neurons. *PLoS Genet.* 15, e1008356. 10.1371/journal.pgen.1008356 31593562 PMC6782096

[DMM049447C157] Xu, F., Kula-Eversole, E., Iwanaszko, M., Hutchison, A. L., Dinner, A. and Allada, R. (2019b). Circadian clocks function in concert with heat shock organizing protein to modulate mutant Huntingtin aggregation and toxicity. *Cell Rep.* 27, 59-70.e4. 10.1016/j.celrep.2019.03.015 30943415 PMC7237104

[DMM049447C158] Yao, Z., Bennett, A. J., Clem, J. L. and Shafer, O. T. (2016). The *Drosophila* clock neuron network features diverse coupling modes and requires network-wide coherence for robust circadian rhythms. *Cell Rep.* 17, 2873-2881. 10.1016/j.celrep.2016.11.053 27974202 PMC5161247

[DMM049447C159] Zar, J. H. (2010). *Biostatistical Analysis*. Prentice Hall.

[DMM049447C160] Zhang, X. and Qian, S.-B. (2011). Chaperone-mediated hierarchical control in targeting misfolded proteins to aggresomes. *Mol. Biol. Cell* 22, 3277-3288. 10.1091/mbc.e11-05-0388 21775628 PMC3172255

[DMM049447C161] Zhang, S., Feany, M. B., Saraswati, S., Littleton, J. T. and Perrimon, N. (2009). Inactivation of *Drosophila* Huntingtin affects long-term adult functioning and the pathogenesis of a Huntington's disease model. *Dis. Model. Mech.* 2, 247-266. 10.1242/dmm.000653 19380309 PMC2675792

[DMM049447C162] Zhou, H., Li, S.-H. and Li, X.-J. (2001). Chaperone suppression of cellular toxicity of huntingtin is independent of polyglutamine aggregation. *J. Biol. Chem.* 276, 48417-48424. 10.1074/jbc.M104140200 11606565

