# Supplementary material for: Hsp40 overexpression in pacemaker neurons delays circadian dysfunction in a Drosophila model of Huntington's disease
Source: Dis Model Mech. 2022 Jun 28;15(6):dmm049447. doi: 10.1242/dmm.049447 (PMC9254228; doi:10.1242/dmm.049447)
Supplement: Supplementary information [file dmm-15-049447-s1.pdf]

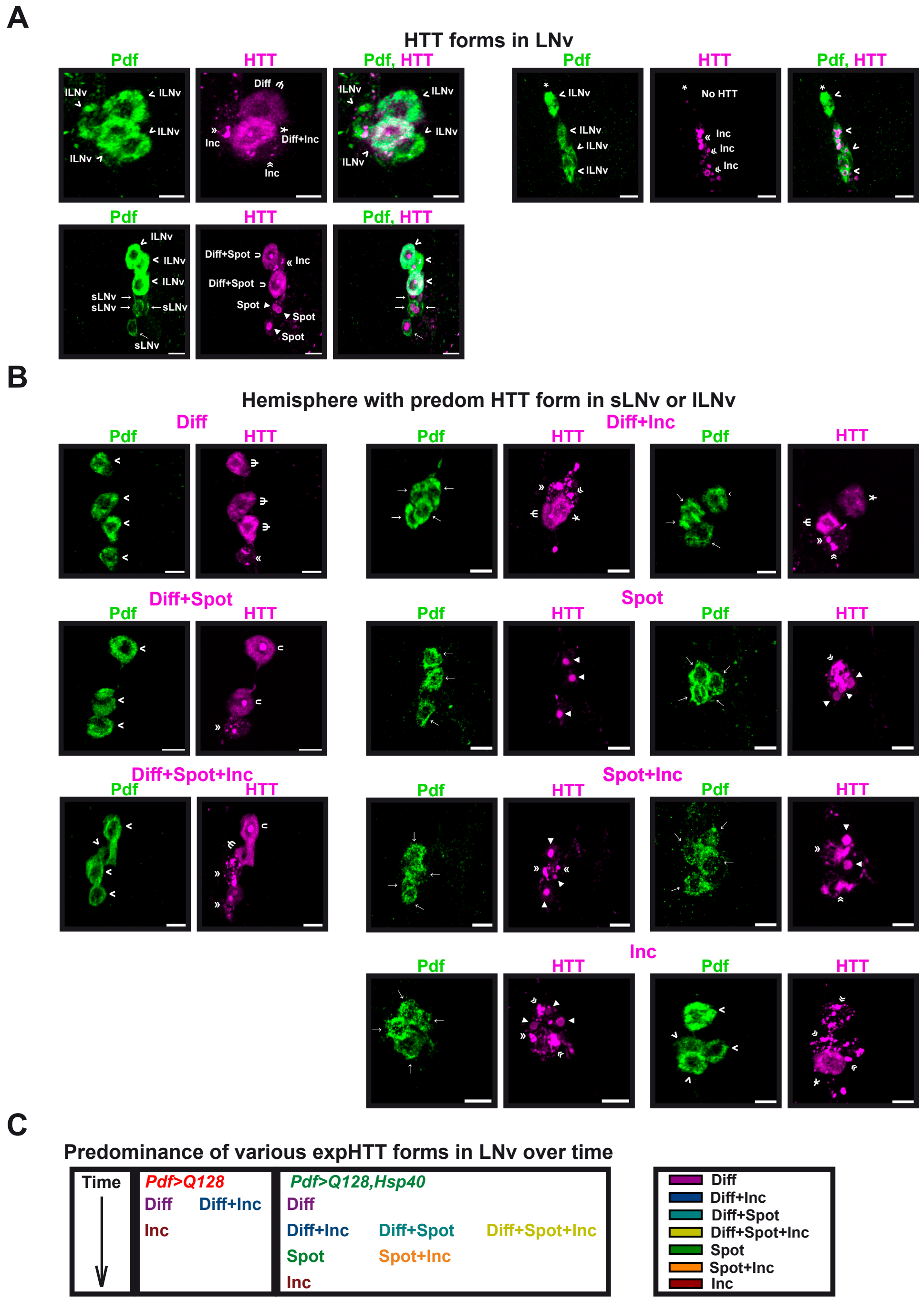

Disease Models & Mechanisms • Supplementary information

Fig. S1. An illustration of the different forms of expHTT detected in LNv. Representative images of adult fly brains stained for Pdf (green) and HTT (magenta) in LNv soma. sLNv soma is indicated by arrows ('→') and lLNv soma by carets ('>'). (A) The various forms of expHTT detected in LNv are shown, namely, diffuse expHTT or Diff ('Ψ' psi), diffuse+inclusions expHTT or Diff+Inc ('¥' symbol), diffuse+spot expHTT or Diff+Spot ('ʊ' upsilon), spot expHTT or Spot ('◀' triangles) and expHTT inclusions or Inc ('«' double carets). The '*' represents an lLNv without expHTT (top-right panels) indicated as NoHTT. (B) The categorisation of hemispheres into seven categories based on the most predominant form of expHTT found in sLNv (or lLNv). The hemispheres are categorised as Diff, Diff+Inc, Diff+Spot, Spot, Diff+Spot+Inc, Spot+Inc and Inc. (C) The predominance of various expHTT forms in LNv over time, based on empirical observations for *Pdf>Q128* and *Pdf>Q128,Hsp40*. The order of appearance and predominance depicted here are for ease of reading and are not to scale are not age-matched events in time across *Pdf>Q128* and *Pdf>Q128,Hsp40*. Scale bars: 10 µm.

Disease Models & Mechanisms • Supplementary information

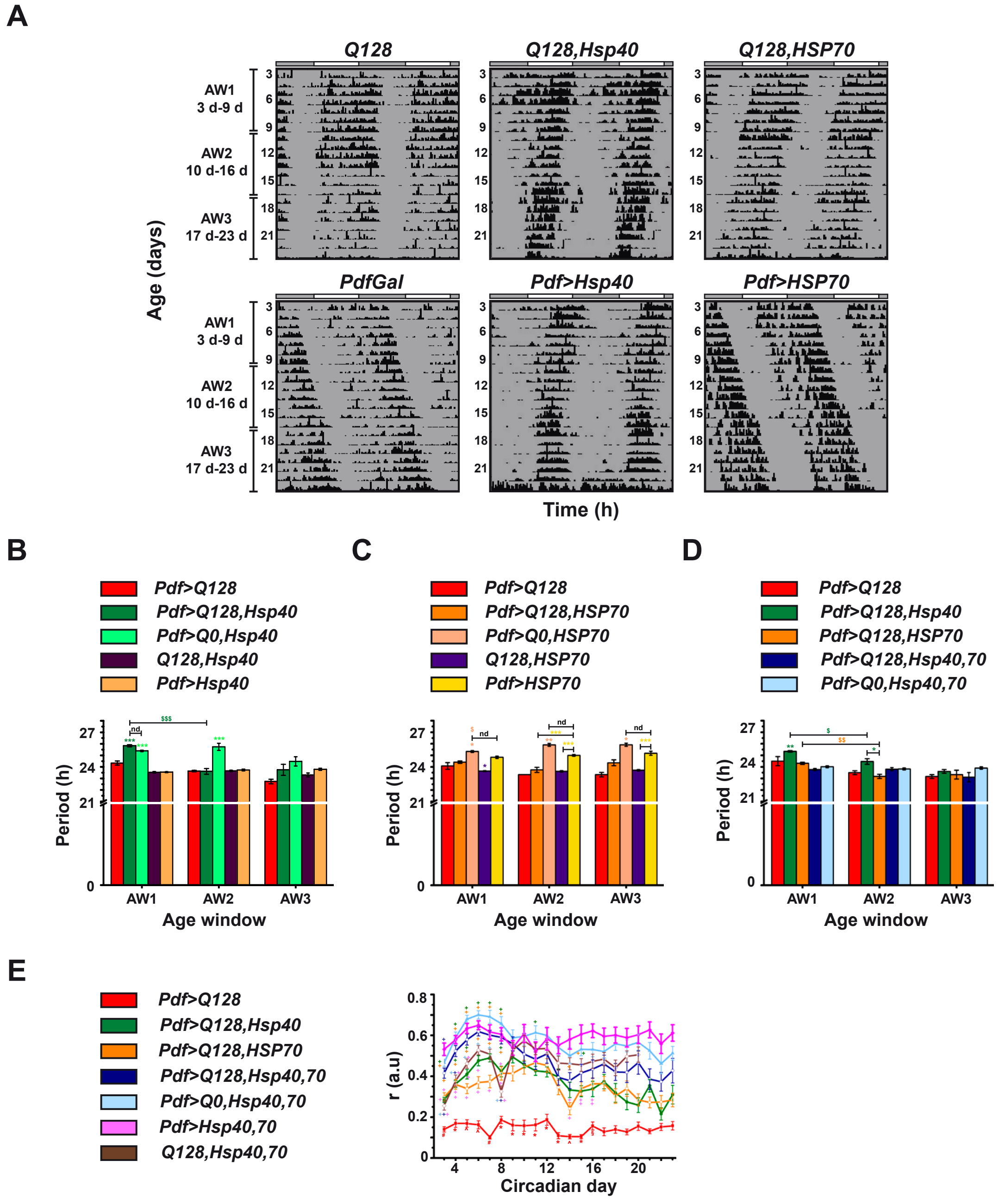

Disease Models & Mechanisms • Supplementary information

Fig. S2. *Pdf>Q128* flies overexpressing Hsp40 or HSP70 show long-period rhythms. (A) Representative double-plotted actograms for flies showing activity data for 21 d (age 3 d-23 d) over three 7 d AWs in DD at 25°C for control genotypes. The white and grey boxes above actograms represent the light and dark phases of the previous LD. (B) - (D) For three different activity runs with the primary experimental genotype being *Pdf>Q128,Hsp40* (B), *Pdf>Q128,HSP70* (C) and *Pdf>Q128,Hsp40,70* (D), comparisons across AWs and between genotypes of the mean period. The following excluded from between-genotype statistical analysis, owing to <10 rhythmic flies: *Pdf>Q128* during AW3 (B) and across AWs (C-D), and *Pdf>Q128,Hsp40* during AW3 analysis (B). Also, because very few rhythmic flies in AW3 in the experimental genotypes of (D), statistical tests were not carried out for AW3. Across all the above panels coloured symbols represents statistically significant differences: coloured '*' indicates age-matched differences of the respective-coloured genotype from all other genotypes or indicated genotype, and coloured '$' indicates differences across age for the respective-coloured genotype. Statistical significance represented by symbols: single, $P<0.05$; double, $P<0.01$; triple, $P<0.001$. nd, not different. (E) Mean '*r*' value (right) comparing genotypes across age for testing the synergistic effect of co-expression on Hsp40 and HSP70 in *Pdf>Q128* flies. Data Post-16 d are omitted from between-genotype statistical tests due to very few surviving flies. The blank in the *Q128,Hsp40,70* line graph between age 21-23 d is due to division of the 21 d data based on the circadian day with a periodicity of ~27 h. Across all panels coloured symbols represents statistically significant differences: red-coloured symbols indicate significant differences at $P<0.05$ of *Pdf>Q128* from '*' all other genotypes, '#' from all genotypes except *Q128,Hsp40,70*, '§' from all genotypes except *Pdf>Q128,Hsp40*, '^' from all genotypes except *Pdf>Q128,HSP70*, and orange '*' of *Pdf>Q128,HSP70* from all other genotypes. Coloured '+' near the error bar of a data-point indicates significant differences at $P<0.05$ of the respective-coloured genotype from the data-point genotype. Error

Disease Models & Mechanisms • Supplementary information

bars are s.e.m. *n* for these analyses are shown in Table S2 as experiment 1 for genotypes of B, under experiment 1 for *HSP70*-related genotypes of C and experiment 4 for *Pdf>Q0* and *Pdf>Q128* of C and as the synergistic effect experiment for D and E.

Disease Models & Mechanisms • Supplementary information

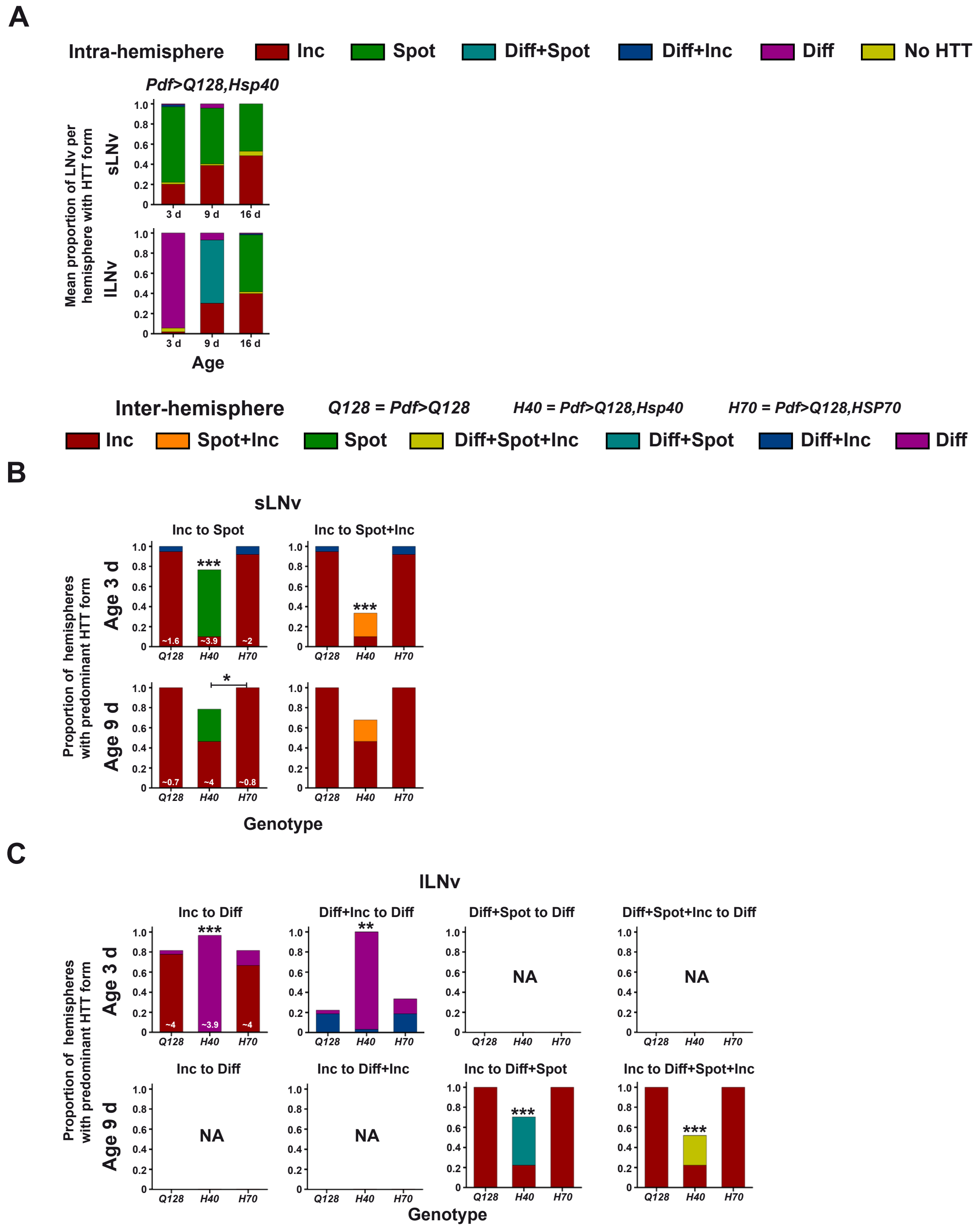

Disease Models & Mechanisms • Supplementary information

Fig. S3. Hemispheres dominated by expHTT inclusion enriched LNv were reduced in favour of diffuse and spot enriched LNv in *Pdf>Q128* flies overexpressing Hsp40. (A) The proportion of sLNv (top) or lLNv (bottom) having different expHTT forms in a hemisphere (intra-hemisphere) averaged across hemispheres are plotted against three ages of *Pdf>Q128,Hsp40* showing the within-hemisphere distribution of expHTT forms. (B) and (C) From the entire set in Fig. 6A, various pair-wise relative hemisphere proportions enriched with expHTT forms in sLNv (B) and lLNv (C) that are statistically significant or (and) are biologically relevant are plotted against the three genotypes for 3 d (top) and 9 d (bottom). At the bottom of some bars, numbers represent the mean number of Pdf^{+} LNv detected for that genotype at that age. '*' indicates significant relative changes in pair-wise proportions of hemispheres enriched in expHTT forms between genotypes at * P<0.05, ** P<0.01 and *** P<0.001. NA, not applicable. *n* for analyses in A are shown in the top-left cell sets of Table S3. *n* for analyses in B and C are shown in the bottom cell sets of Table S3.

Disease Models & Mechanisms • Supplementary information

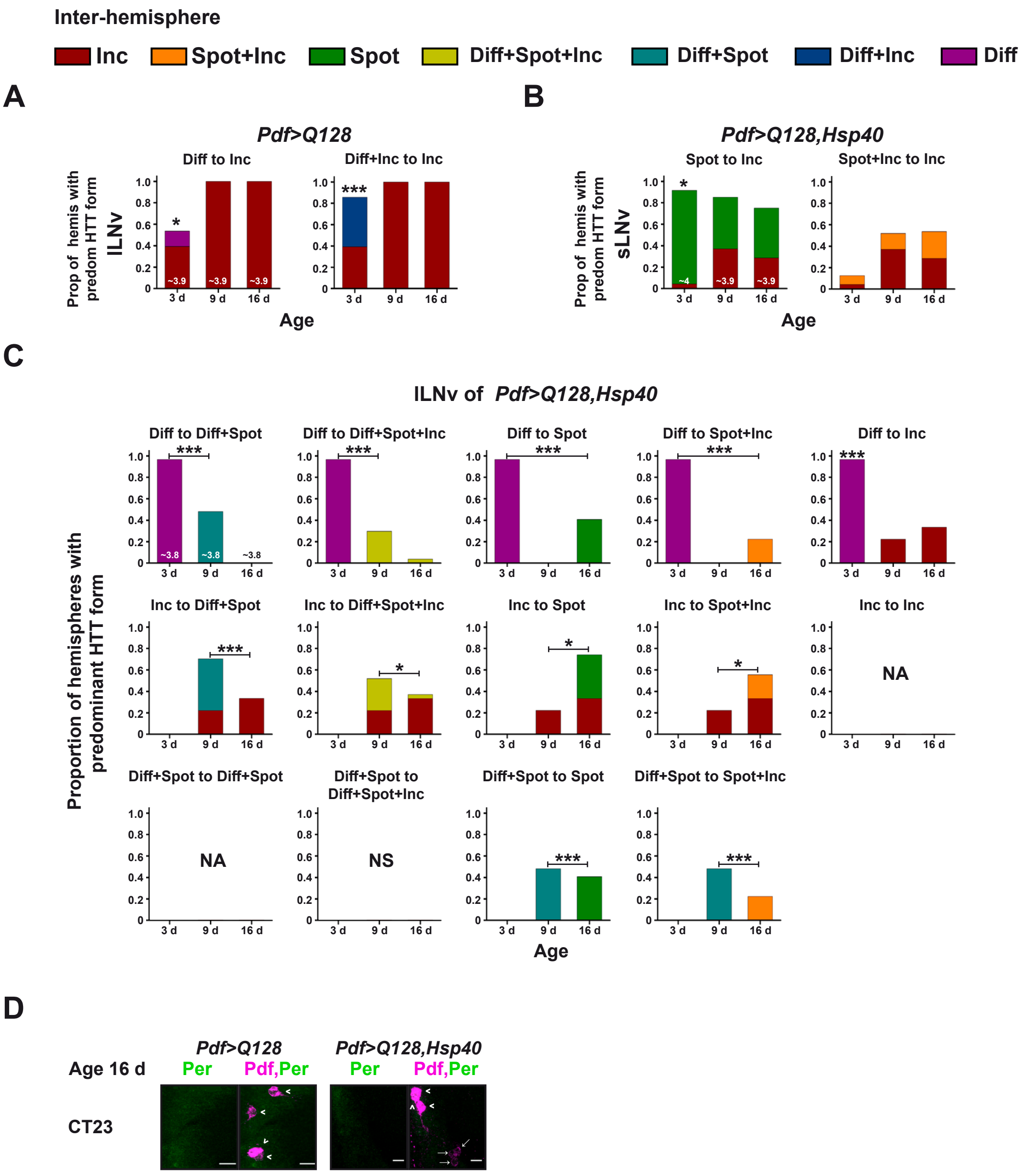

Disease Models & Mechanisms • Supplementary information

Fig. S4. Most *Pdf>Q128* flies overexpressing Hsp40 possess hemispheres with expHTT spot form enriched LNv. (A-C) From the entire set in Fig. 6C, various pair-wise relative hemisphere proportions enriched with expHTT forms in lLNv (A and C) and sLNv (B) that are statistically significant or (and) are biologically relevant are plotted against age for the genotypes *Pdf>Q128* (A) and *Pdf>Q128,Hsp40* (B and C). At the bottom of some bars, numbers represent the mean number of Pdf^{+} LNv detected for that genotype at that age. '*' indicates significant relative changes in pair-wise proportions of hemispheres enriched in expHTT forms between genotypes at '*' $P<0.05$, '**' $P<0.01$ and '***' $P<0.001$. NA, not applicable; NS, not significant. (D) Representative images of 16 d old adult fly brains of *Pdf>Q128* and *Pdf>Q128,Hsp40* stained for Per (green) and Pdf (magenta) in LNv at CT23. Arrows ('→') indicate sLNv and carets ('>') lLNv. Scale bars: 10 µm. *n* for analyses in A, B and C are shown in the top-left cell sets of Table S3.

Disease Models & Mechanisms • Supplementary information

Table S1. A genetic screen for modifiers of arrhythmicity of expHTT expressing flies. The genetic modifiers are grouped according to their most well-known function or protein family. OE, overexpression; DR, down-regulation. Each modifier genotype is compared against its respective *circadian driver-Q128* genotype (placed at the top of the table), i.e. either the recombinant *w;PdfGal4-Q128/+* (denoted as *w;Pdf-Q128/+;+*) or *w;PdfGal4/Q128;+* or *w;TimGal4/Q128;+*. '*' indicates significant differences from respective controls at * *P*<0.05, ** *P*<0.01, *** *P*<0.001.

Gene	Description	Modification	Modifier Line Source	Genotype (s)	% Rhythmicity
**Huntingtin**					
hHTT	Human huntingtin with 548aa containing 128 Q repeats		Lee et al., 2000	***w;Pdf-Q128/+;+***	54.84
				w;PdfGal4/Q128;+	20
				w;TimGal4/Q128;+	0
dhtt	*Drosophila* huntingtin	OE	Zhang et al., 2009	***w;Pdf-Q128/UAS-dhtt;+***	50
dhtt	*Drosophila* huntingtin with 82aa	OE	Mugat et al., 2008	***w;Pdf-Q128/+;UAS-dhtt82aa/+***	60
				w;PdfGal4/Q128;UAS-dhtt82aa/+	6.25
				w;TimGal4/Q128;UAS-dhtt82aa/+	0
dhtt	*Drosophila* huntingtin with 620aa	OE	Mugat et al., 2008	***w;Pdf-Q128/+;UAS-dhtt620aa/+***	31.25
				w;PdfGal4/Q128;UAS-dhtt620aa/+	12.5
				w;TimGal4/Q128;UAS-dhtt620aa/+	0
dhtt	*Drosophila* huntingtin	DR	GD 36204	***w;Pdf-Q128/UAS-dhttRNAi/+;+***	62.5
dhtt	*Drosophila* huntingtin	DR	GD29532	***w;Pdf-Q128/+;UAS-dhttRNAi/+***	53.33
				w;PdfGal4/Q128;UAS-dhttRNAi/+	12.5
				w;TimGal4/Q128;UAS-dhttRNAi/+	6.25
Hip14	*Drosophila* Huntingtin interacting protein	OE	Bl 17109	***w;Pdf-Q128/+;UAS-Hip14/+***	73.33

Disease Models & Mechanisms • Supplementary information

Gene	Description	Modification	Modifier Line Source	Genotype (s)	%Rhythmicity
**Heat Shock Proteins (HSP)**					
Hsp23	*D.melanogaster* Hsp23	OE	Bl 30541	***w;Pdf-Q128/UAS-Hsp23;+***	86.67*
HSAP/ JB1/ DNAJB1	Human DNAJB1 (Hsp40 homolog, sub-family B, member 1)	OE	Bl 53730	***w;Pdf-Q128/ UAS-DNAJB1;+***	93.75**
DnaJ1k/ dhdJ1	*D.melanogaster* DnaJ-like-1 or Hsp40, droj1 (member of J/Hsp40 co-chaperone family; provides specificity to Hsp70 chaperones)	OE	Bl 30553	***w;Pdf-Q128/+; UAS-DnaJ1k/+***	93.75**
HSPA1L/ HSP70/ HSAP	Human heat shock 70kDa protein 1-like or Hsp70	OE	Bl 7454	***w;Pdf-Q128/+; UAS-HSPA1L/+***	93.75*
				w;PdfGal4/Q128; UAS-HSPA1L/+	100***
				w;TimGal4/Q128; UAS-HSPA1L/+	43.75**
Hsc70-3	*D.melanogaster* heat shock 70-kDa protein cognate 3 or BiP or Hsc3 or Grp78	OE	Bl 5843	***w;Pdf-Q128/+; UAS-Hsc70-3WT/+***	43.75
Hsc70-3	*D.melanogaster* Hsc70-3 with a disrupted ATP binding site	DR	Bl 5842	***w;Pdf-Q128/+; UAS-Hsc70-3.K97S/+***	80
Hsc70-4	*D.melanogaster* heat shock protein cognate 4 or Hsc4 or BAP74 or scattered	DR	Bl 7453	***w;Pdf-Q128/+; UAS-Hsc70-4K71S/+***	100**
Hsf	*D.melanogaster* Heat shock factor 1 or Hsf1 or Dm-Hsf	DR	Bl 27070	***w;Pdf-Q128/+; UAS-Hsf-RNAi/+***	58.33
Hsf	*D.melanogaster* Heat shock factor 1 or Hsf1 or Dm-Hsf	DR	GD 48692	***w;Pdf-Q128/ UAS-Hsf-RNAi/+***	62.5
Hdj1/ Hsp40	*D.melanogaster* dHdj1/ Hsp40	DR	GD 31271	***w;Pdf-Q128/+; UAS-Hdj1-RNAi/+***	60
Hsp70Aa	*D.melanogaster* Heat-shock-protein-70Aa	DR	GD 41748	***w;Pdf-Q128/+; UAS-Hsp70Aa-RNAi/+***	68.75
Hsp70Bb	*D.melanogaster* Heat-shock-protein-70Bb	DR	GD 36640	***w;Pdf-Q128/+; UAS-Hsp70Bb-RNAi/+***	75

Gene	Description	Modification	Modifier Line Source	Genotype (s)	% Rhythmicity
**Histone Deacetylases**					
Hdac3	*D.melanogaster* histone deacetylase involved in chromatin silencing	OE	Bl 32248	***w;Pdf-Q128/UAS-Hdac3;+***	31.25
Hdac6	*D.melanogaster* cytosolic deacetylase that functions as a key modulator of proteostasis	OE	Bl 51181	***w;Pdf-Q128/+;UAS-Hdac6/+***	56.25
Hdac3	*D.melanogaster* histone deacetylase	DR	Bl 34778	***w;Pdf-Q128/+;UAS-Hdac3RNAi/+***	50
**Autophagy**					
atg1/ unc-51/ DK-4	*D.melanogaster* Autophagy-related 1; a protein kinase, functions in regulation of autophagy	OE	Bl 51654	***w;Pdf-Q128/UAS-atg1;+***	60
			Bl 51655	***w;Pdf-Q128/+;UAS-atg1/+***	43.75
atg5	*D.melanogaster* Autophagy-related 5; has Atg8 ligase activity	OE	Bl 59848	***w;Pdf-Q128/+;UAS-atg5/+***	87.5*
atg8a/ LC3	*D.melanogaster* Autophagy-related 8a; has roles in autophagosome formation, maintenance of neuromuscular function and normal lifespan	OE	Bl 52005	***w;Pdf-Q128/UAS-atg8a;+***	88.24*
			Bl 51656	***w;Pdf-Q128/+;UAS-atg8a/+***	87.5*
**Apoptosis**					
Iap2/ Diap-2/ Diha	*D.melanogaster* Death-associated inhibitor of apoptosis 2; ubiquitin E3-ligase activity	DR	Bl 34776	***w;Pdf-Q128/+; UAS-Iap2RNAi /+***	62.5
dark/ ark/ hac-1/ Apaf-1	*D.melanogaster* Death-associated Apaf1-related killer (Dark); an essential component of the apoptosome	DR	KK 104215	***w;Pdf-Q128/UAS-darkRNAi;+***	35.71

Disease Models & Mechanisms • Supplementary information

Table S2. Number of surviving flies in AW2 and AW3 for locomotor activity experiments

		Sample Sizes				Sample Sizes	
**Genotype**	**Exper-iment**	**AW2**	**AW3**	**Genotype**	**Exper-iment**	**AW2**	**AW3**
Pdf>Q0,Hsp40	1	31	18	***Pdf>Q128,Hsp40***	1	32	18
	2	30	24		2	32	31
	3	27	22		3	32	29
	4	31	31		4	32	31
	5	31	28		5	31	30
Pdf>Q0	1	29	9	***Pdf>Q128***	1	30	21
	2	31	30		2	31	31
	3	24	24		3	28	26
	4	30	30		4	31	29
	5	16	12		5	27	23
Q0,Hsp40	1	25	4	***Q128,Hsp40***	1	29	11
	2	30	26		2	32	31
	3	15	0		3	13	0
Pdf>Q0,HSP70	1	28	24	***Pdf>Q128,HSP70***	1	32	32
	2	29	23		2	29	26
	3	23	17		3	32	28
Q0,HSP70	1	22	21	***Q128,HSP70***	1	27	25
Pdf>Hsp40	1	32	13	***Pdf>HSP70***	1	28	23
	2	30	29				
**Synergistic Effect Experiment**							
Pdf>Q128,Hsp40,70		22	17	***Pdf>Q128***		27	28
Pdf>Q0,Hsp40,70		26	21	***Pdf>Q128,Hsp40***		21	21
Pdf>Hsp40,70		22	19	***Pdf>Q128,HSP70***		30	28
Q128,Hsp40,70		28	24				

Disease Models & Mechanisms • Supplementary information

Table S3. Number of hemispheres per genotype per age used for quantification of cellular features. Top: For Per oscillations, flies were dissected around CT23 and CT11 at different ages: all the five genotypes at 3 d and *Pdf>Q0,Hsp40* and *Pdf>Q128,Hsp40* also at 9 d (*n* for Fig. 7D). For Pdf^+ and Per^+ LNv numbers, samples at CT23 (top-left) were used (*n* for Fig. 4; Fig. 7B,C). Bottom: For quantification of expHTT inclusions and expHTT forms in *Pdf>Q128*, *Pdf>Q128,Hsp40* and *Pdf>Q128,HSP70* across 3 d and 9 d (*n* for Fig. 6A,B,E; Fig. S3B,C). For comparisons of expHTT forms across 3 d, 9 d and 16 d for in *Pdf>Q128* and *Pdf>Q128,Hsp40*, samples at CT23 (top-left) were used (*n* for Fig. 5C; Fig. 6C,D; Fig. S3A; Fig. S4A-C).

**Number of hemispheres/genotype/time-point/age**					
	**CT23**			**CT11**	
**Genotype**	**3 d**	**9 d**	**16 d**	**3 d**	**9 d**
Pdf>Q128	28	29	23	30	
Pdf>Q0,Hsp40	21	26	27	27	27
Pdf>Q128,Hsp40	26	29	28	25	25
Pdf>Q0,HSP70	26	18		31	
Pdf>Q128,HSP70	30	23		27	

**expHTT Inclusions**		
**Genotype**	**3 d**	**9 d**
Pdf>Q128	27	20
Pdf>Q128,Hsp40	30	30
Pdf>Q128,HSP70	27	25

Disease Models & Mechanisms • Supplementary information
